# Prophylaxis of graft-versus-host-disease: Systematic evidence-based recommendations of the Brazilian association of hematology, hemotherapy, and cell therapy (ABHH) and the Brazilian society of cellular therapy and bone marrow transplantation (SBTMO)

**DOI:** 10.1016/j.htct.2026.106494

**Published:** 2026-07-09

**Authors:** Afonso C. Vigorito, Carmino A. de Souza, Nelson Hamerschlak, Vaneuza A.M. Funke, Maria Claudia R. Moreira, Adriana Seber, Renato Luiz Guerino Cunha, Wanderley Bernardo, Fernando B. Duarte

**Affiliations:** a Hospital das Clínicas/Hemocentro da Universidade Estadual de Campinas, UNICAMP, Campinas, SP, Brazil; b Faculdade de Ciências Médicas, Universidade Estadual de Campinas, Unicamp, Campinas, São Paulo, Brazil; c Hospital Albert Einstein, São Paulo, SP, Brazil; d Complexo Hospital de Clínicas, Universidade Federal do Paraná, Curitiba, PR, Brazil; e Hospital Nossa Senhora das Graças, Curitiba, PR, Brazil; f Instituto Nacional de Câncer, INCA, Rio de Janeiro, RJ, Brazil; g Serviço de Hematologia, Hospital Universitário Clementino Fraga Filho, Universidade Federal do Rio de Janeiro, Rio de Janeiro, RJ, Brazil; h Hospital Samaritano, São Paulo, SP, Brazil; i Cellular Therapy Program, Oncoclínicas São Paulo, São Paulo, SP, Brazil; j Faculdade de Medicina da Universidade de São Paulo, USP, São Paulo, SP, Brazil; k Hospital Universitário Walter Cantidio, Universidade Federal do Ceará, Fortaleza, CE, Brazil

**Keywords:** Bone marrow transplantation, Graft vs host disease, Practice guideline, Systematic review, Prevention and control

## Abstract

Graft-versus-host disease, involving from 20%–80% of patients, is one of the main complications of allogeneic hematopoietic cell transplantation contributing significantly to mortality and morbidity. The methodological basis of these graft-versus-host disease prophylaxis guidelines for allogeneic hematopoietic cell transplantation, developed by the Brazilian Association of Hematology, Hemotherapy, and Cell Therapy (ABHH) and the Brazilian Society of Cellular Therapy and Bone Marrow Transplantation (SBTMO), is a review which applied the GRADE process to eleven PICO (population, intervention, comparator, and outcome) questions. These questions address graft-versus-host disease prophylaxis in allogeneic hematopoietic cell transplantation across various settings, including myeloablative and non-myeloablative/reduced-intensity conditioning. The scope encompasses related matched, unrelated matched or mismatched, and haploidentical donors, utilizing both bone marrow and peripheral blood as stem-cell sources. The combination of a calcineurin inhibitor (e.g., cyclosporine or tacrolimus) plus methotrexate or mycophenolate mofetil (MMF) has been the standard graft-versus-host disease prophylaxis regimen for allogeneic hematopoietic cell transplantation. The excellent results of post-transplant cyclophosphamide in haploidentical related donor hematopoietic cell transplantation have led to broader use in human leukocyte antigen-(mis)matched related and unrelated donor grafts. Sirolimus, low-dose antithymocyte globulin (4–6 mg/kg) and more recently abatacept are other drugs used. Hence, this review is meant to be a useful reference tool for clinicians who are dealing with this complex complication.

## Introduction

Graft-versus-host disease (GvHD) can occur after allogeneic hematopoietic cell transplantation (allo-HCT) when immune cells from a donor (the graft) initiate an immune reaction against a transplant recipient (the host). According to National Institutes of Health (NIH) consensus criteria, acute GvHD (aGvHD) and chronic GvHD (cGvHD) are multisystem disorders that are distinguished by clinical findings [1, 2, 3].

Despite prophylactic treatment with immunosuppressive agents, 20%–80% of allo-HCT recipients develop aGvHD [4]. The main risk factors for aGvHD are human leukocyte antigen (HLA)-mismatching between donor and recipient, use of an unrelated donor, gender disparity (specifically female donor to male recipient), myeloablative conditioning, total-body-irradiation-based conditioning, progenitor stem cell source (peripheral blood > bone marrow), and older donor age [5, 6].

With a prevalence of 30%–70% among allo-HCT recipients, cGvHD remains the main cause of long-term post-transplant morbidity and mortality in this population [7]. Risk factors associated with cGvHD are hematopoietic cell transplantation (HCT) with HLA-mismatched or unrelated donor, use of a female donor for a male recipient, grafting with mobilized blood, prior aGvHD, and older donor or recipient age [5].

The success of allo-HCT depends on the prophylaxis of GvHD without relapse of the underlying disease, which can occur when the alloimmune response is blunted by excessive immunosuppressive GvHD prophylaxis [8].

The combination of a calcineurin inhibitor (CNI: e.g., cyclosporine or tacrolimus) plus methotrexate or MMF has been the standard GvHD prophylaxis regimen for allo-HCT. However, the excellent results of post-transplant cyclophosphamide (PTCy) in haploidentical related donor HCT have led to broader use in cases of HLA-(mis)matched related and unrelated donor grafts [9, 10, 11, 12]. Sirolimus, low-dose antithymocyte globulin (ATG) (4–6 mg/kg), and more recently abatacept are other drugs used [13].

These evidence-based guidelines, developed using the PICO (Population, Intervention, Comparator, and Outcome) methodology, address GvHD prophylaxis in allo-HCT performed with non-myeloablative/reduced-intensity or myeloablative conditioning regimens and using related matched, unrelated matched or mismatched, and haploidentical donors, with bone marrow or peripheral blood as the progenitor stem cell source.

## Methods

The methodological basis of these GvHD prophylaxis guidelines for allo-HCT, developed by the Brazilian Association of Hematology, Hemotherapy, and Cell Therapy (ABHH) and the Brazilian Society of Cellular Therapy and Bone Marrow Transplantation (SBTMO), is a formal and explicit systematic review that applied the GRADE process to eleven PICO (Population, Intervention, Comparator, and Outcome) questions. For all questions there are common eligibility criteria related to study design (observational or experimental studies), search period (no restriction), language (Portuguese, Spanish, English) and the inclusion of full texts or abstracts with data. The other eligibility criteria varied by question and were directly related to the specified patients, interventions, comparisons, and outcomes. In general, the methodology followed the steps outlined below:

• **Information Sources:** The following electronic databases were searched: Medline, Embase, Clinical Trials, Google Scholar. Additionally, a manual search of relevant reference lists was performed. The following search terms were used:

#1 (Bone Marrow Transplantation OR Bone Marrow Grafting)

#2 (Hematopoietic Stem Cell Transplantation OR Peripheral Blood Stem Cell Transplantation)

#3 (Graft vs Host Disease OR Graft-Versus-Host Disease OR Graft Versus Host Disease OR Runt Disease OR Graft-vs-Host Disease OR Graft-vs-Host Diseases)

#4 (Abatacept OR “Antilymphocyte Antibody” OR “Antilymphocyte Globulin” OR “Antilymphocyte Globulins” OR “Antilymphocyte Immunoglobulin” OR “Antilymphocyte Serum” OR “Anti-T lymphocyte globulin” OR Antithymoglobulin OR “Antithymocyte Globulin” OR “Anti-Thymocyte Globulin” OR “Anti Thymocyte Globulin” OR “Antithymocyte immunoglobulin” OR “Anti-human thymocyte globulin” OR “Anti-T-cell globulin” OR ATGAM OR ATG OR ATLG OR “Calcineurin Antagonists” OR “Calcineurin Blockers” OR “Calcineurin Inhibitor” OR “Calcineurin Inhibitors” OR Ciclosporin OR Cy OR Cyclosporin OR Cyclosporine OR Cyclosporins OR Cyclophosphamide OR CsA OR CyA OR CSP OR Everolimus OR Methotrexate OR “Mycophenolic Acid” OR “Mycophenolate Mofetil” OR PTCy OR Sirolimus OR Tacrolimus OR Thymoglobulin)

#5 (“Comparative study” OR “Comparative studies” OR ((clinical [Title/Abstract] AND trial [Title/Abstract]) OR clinical trials as topic [MeSH Terms] OR clinical trial [Publication Type] OR random* [Title/Abstract] OR random allocation [MeSH Terms] OR therapeutic use [MeSH Subheading]) OR “Epidemiologic methods” OR “Multicenter study”)

#6 = #1 OR #2 OR #3

#7 = #6 AND #4 AND #5 (global search)

• **Data Extraction and Presentation of Results:** The extracted outcomes were clinical rather than intermediate and included aGvHD, cGvHD, overall survival (OS), relapse, non-relapse mortality (NRM), disease-free survival (DFS), GvHD-Free and relapse-free survival (GRFS), event-free survival (EFS: such as disease, progression, graft failure, death), invasive fungal disease, bacterial infections, and cytomegalovirus and Epstein-Barr virus reactivation. Results were expressed as the absolute risk of events or outcomes in each group, risk differences, 95% confidence intervals (95% CI), and the number needed to treat (NNT) or the number needed to harm (NNH).
• **Risk-bias assessment and quality of evidence:** The risk of bias of the studies was assessed using the expanded Rob 2 instrument. The variables included were randomization, allocation concealment, blinding (double-blinding and blinded outcome assessment), prognostic characteristics, losses, outcomes (timing, appropriateness, and measurement), intention-to-treat analysis, sample calculation, and early interruption. The quality of evidence was assessed using the GRADE classification as very low, low, moderate, or high based on the risk of bias (when meta-analysis was not possible).
• **Data analysis, evidence synthesis and recommendation:** If there were common outcomes reported by the selected works, these were aggregated in a meta-analysis using software and utilizing a consistent effect measure (difference in risk and 95% CI), a priori fixed or random model (depending on the heterogeneity calculated by I2). A random-effects model was used when the I2 exceeded 50%, otherwise a fixed-effect model was applied. Finally, a summary of findings (SoF) table was generated to support the recommendations, which were designed to answer the initial clinical questions.
• All the steps were monitored, evaluated, and validated by a panel of experts in allo-HCT from the ABHH and SBTMO. Disagreements were resolved by consensus, and no formal adjudication by a third reviewer was required.

## Results

**Figure 1:**
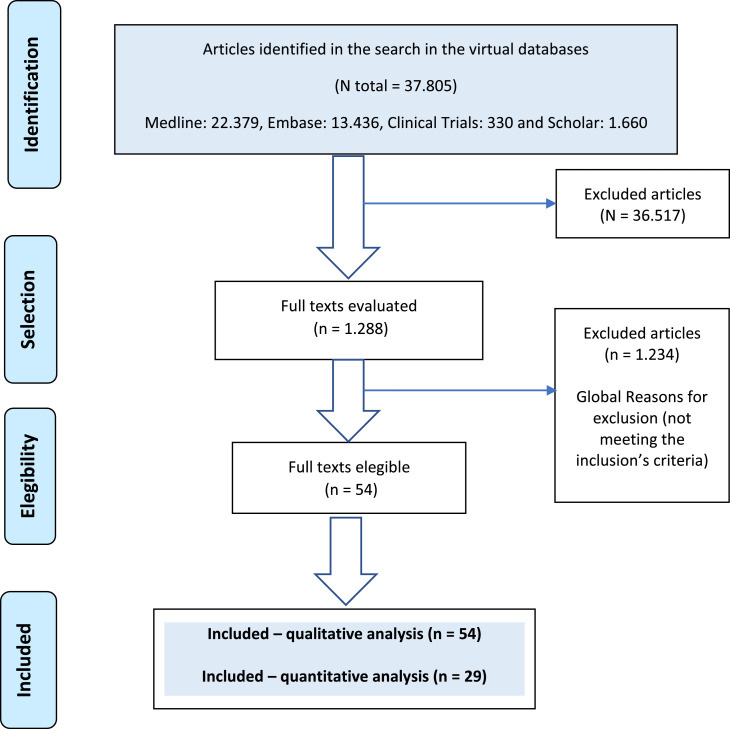
PRISMA flow diagram of study selection for graft-versus-host disease prophylaxis.

The search process retrieved a total of 37,805 articles (Medline: 22,379; Embase: 13,436; ClinicalTrials.gov: 330; and Google Scholar: 1660). Based on the inclusion criteria, 1288 articles were selected for full-text or abstract review. Ultimately, 54 studies were selected to support the recommendations. Data from 29 publications were aggregated in the meta-analyses for Questions 6-8. Meta-analyzes was performed for Questions 3-8 (Figure 1). Reasons for exclusion depended on the specific criteria of each question and are presented as general reasons for not meeting the inclusion criteria.

**CLINICAL QUESTION 1**

What are the efficacy and risks of the cyclosporine plus methotrexate regimen in patients undergoing myeloablative allogeneic hematopoietic cell transplantation (allo-HCT) with related or unrelated matched donors?

**Evidence synthesis**

• Tacrolimus plus methotrexate showed superiority over cyclosporine plus methotrexate in the prophylaxis of Grade II–IV aGvHD with no significant difference in the DFS or OS in patients with non-advanced disease [14].
• The combination of tacrolimus plus methotrexate was more effective than cyclosporine plus methotrexate for the prophylaxis of aGvHD, without any significant increase in graft failure, relapse, or toxicity [15].
• GvHD prophylaxis with cyclosporine plus MMF following a myeloablative allogeneic preparative regimen was associated with faster hematopoietic engraftment and a decreased incidence of mucositis compared to cyclosporine plus methotrexate. The incidence of aGvHD and survival rates remained comparable [16]. With a longer follow-up, there were no significant differences in infection rates or late mortality [17].

**Recommendation**

For patients undergoing myeloablative allo-HCT with related or unrelated matched donors, the use of cyclosporine plus methotrexate is a therapeutic option for reducing the risk of aGvHD. However, this has not been directly associated with a reduction in mortality.

**Quality of Evidence:** Very Low

**CLINICAL QUESTION 2**

What are the efficacy and risks of the tacrolimus plus methotrexate regimen in patients undergoing myeloablative allogeneic hematopoietic cell transplantation (allo-HCT) with related or unrelated matched donors?

**Evidence synthesis**

The use of tacrolimus plus methotrexate had the following effects compared with:

***Cyclosporine plus Methotrexate***

• Tacrolimus plus methotrexate showed superiority over cyclosporine plus methotrexate in the prophylaxis of Grade II-IV aGvHD with no significant difference in the DFS or OS in patients with non-advanced disease [14].
• The combination of tacrolimus plus methotrexate was more effective than cyclosporine plus methotrexate for the prophylaxis of aGvHD, without any significant increase in graft failure, relapse, or toxicity [15].

***Tacrolimus plus mini-Methotrexate and Mycophenolate Mofetil***

• Compared with the full-methotrexate regimen, mini-methotrexate plus MMF was associated with no significant difference in Grade II-IV aGvHD and a more favorable toxicity profile; however, a higher incidence of Grade III-IV aGvHD was seen with mini-methotrexate plus MMF. Patients who received the mini-methotrexate plus MMF regimen had less Grade 3 or 4 mucositis and faster engraftment. There were no significant differences in moderate-to-severe cGvHD at one year or infection rates. Patients who received the mini-methotrexate plus MMF regimen had less nephrotoxicity and respiratory failure. There were no significant differences in the one-year relapse and OS rates [18].

***Tacrolimus plus Sirolimus***

• Patients who received sirolimus plus tacrolimus had significantly less moderate-severe cGvHD and late aGvHD. Patients who received sirolimus plus tacrolimus had lower prednisone exposure and earlier discontinuation, but there was no significant difference in the total discontinuation of immunosuppression [19].
• There was no significant difference in the GvHD-free survival. The sirolimus plus tacrolimus regimen can be considered in patients undergoing total-body-irradiation-based conditioning who are at higher risk of oropharyngeal mucositis, and in patients in whom timely engraftment is required after appropriate screening for risks of excessive hepatotoxicity are excluded [20].
• Sirolimus plus tacrolimus prevents Grade II-IV aGvHD and moderate-severe cGvHD more effectively than methotrexate plus tacrolimus [21].

**Recommendation**

For patients undergoing myeloablative allo-HCT with related or unrelated matched donors, the tacrolimus plus methotrexate regimen is a therapeutic option to reduce the risk of aGvHD and cGvHD, although this has not been directly associated with a reduction in mortality.

**Quality of Evidence:** Very Low

**CLINICAL QUESTION 3**

What are the efficacy and risks of a regimen using a calcineurin inhibitor (CNI) and MMF in patients undergoing myeloablative allogeneic hematopoietic cell transplantation with related or unrelated matched donors?

**Evidence Synthesis**

The CNI plus MMF and CNI plus methotrexate regimens were compared. Quantitative data were presented as the number of events for each outcome, and the absolute risk for each group (MMF and methotrexate) was calculated as the ratio between the number of events and the total number of patients. The results of the meta-analysis expressed the difference in risk between the two groups compared as well as the 95% confidence interval (95% CI).

• There was no significant difference in the risk of aGvHD when comparing the combination of CNI plus MMF versus the combination with methotrexate, regardless of the use of tacrolimus or cyclosporine (Supplementary Figure 1). Very low quality of evidence [16,18,22].
• There was no significant difference in the risk of cGvHD when comparing the combination of CNI plus MMF versus the combination with methotrexate, regardless of the use of tacrolimus or cyclosporine (Supplementary Figure 2). Low quality of evidence [16,18].
• There was no significant difference in mortality when comparing the combination of CNI plus MMF versus the combination with methotrexate, regardless of the use of tacrolimus or cyclosporine (Supplementary Figure 3). Low quality of evidence [16,18,22].
• There was no significant difference in relapse when comparing the combination of CNI plus MMF versus the combination with methotrexate, regardless of the use of tacrolimus or cyclosporine (Supplementary Figure 4). Very low quality of evidence [16,18].
• There was a 30% reduction in the risk of mucositis (95% CI: 15%–45%) with the use of CNI plus MMF compared to the methotrexate combination (Supplementary Figure 5). The number needed to treat to prevent one case of mucositis was 3 (NNT: 3). In the subgroup analysis, the risk reduction was greater for the cyclosporine-based regimen (44%) than for the tacrolimus-based combination (24%) (Supplementary Figure 5). Moderate quality of evidence [16,18].
• There was no significant difference in the risk of infection when comparing the combination of CNI plus MMF versus the combination with methotrexate, regardless of the use of tacrolimus or cyclosporine (Supplementary Figure 6). Low quality of evidence [16,18].
• There was a 20% reduction in the risk of toxicity, specifically nephrotoxicity and respiratory failure (95% CI: 4%–36%) with the use of tacrolimus plus MMF compared to tacrolimus plus methotrexate (Supplementary Figure 7). The number needed to treat to prevent one toxicity event was 5 (NNT: 5). Moderate quality of evidence [18].

**Recommendations**

In patients undergoing myeloablative allo-HCT with related or unrelated matched donors, the efficacy of GvHD prophylaxis using a regimen containing CNI (cyclosporine or tacrolimus) plus MMF is comparable to the combination with methotrexate in respect to aGvHD, cGvHD, mortality, relapse, and infection rate. However, the combination with MMF can be recommended due to lower toxicity (nephrotoxicity and respiratory failure), particularly with tacrolimus and the lower incidence of mucositis (regardless of cyclosporine and tacrolimus), when compared to the combination with methotrexate.

**Quality of Evidence:** varied between very low and moderate, depending on the analyzed outcome.

**CLINICAL QUESTION 4**

What are the efficacy and risks of regimens using low-dose (<6 mg/kg) or high-dose (≥6 mg/kg) rabbit anti-thymocyte globulin in patients undergoing allogeneic hematopoietic cell transplantation using matched sibling or unrelated peripheral blood donors?

**Evidence Synthesis**

Quantitative data were presented as the number of events for each outcome, and the absolute risk for each group, intervention (ATG) and control (no ATG), was calculated as the ratio of the number of events to the total number of patients. The results of the meta-analysis expressed the risk difference for the outcome between the two groups, along with the 95% CI. A subgroup analysis was performed based on the doses used: low-dose (<6 mg/kg) or high-dose (≥6 mg/kg).

• In patients undergoing allo-HCT, the use of rATG (regardless of dose) in combination with standard GvHD prophylaxis, compared with standard prophylaxis alone, was associated with a 15% reduction in the risk of aGvHD (95% CI: 10%–20%). In the subgroup analysis, the reduction risk ranged from 13% (low-dose) to 17% (high-dose) (Supplementary Figure 8) [23, 24, 25, 26, 27, 28, 29]. Moderate quality of evidence.
• In patients undergoing allo-HCT, the use of rATG in combination with standard GvHD prophylaxis, compared with standard prophylaxis alone, was associated with an 18% reduction in the risk of cGvHD (95% CI: 13%–23%); however, this effect was significant only in the high-dose subgroup. In the subgroup analysis, the reduction ranged from 9% (low-dose) to 25% (high-dose) (Supplementary Figure 9) [23, 24, 25, 26, 27, 28, 29]. Low quality of evidence.
• In patients undergoing allo-HCT, the use of rATG (regardless of dose) in combination with standard GvHD prophylaxis, compared with standard prophylaxis alone, showed no significant difference in OS (Supplementary Figure 10) [23, 24, 25, 26, 27, 28, 29]. Low quality of evidence.
• In patients undergoing allo-HCT, the use of rATG (regardless of dose) in combination with standard GvHD prophylaxis, compared with standard prophylaxis alone, showed no significant difference in DFS (Supplementary Figure 11) [23, 24, 25, 26, 27,29]. Very Low quality of evidence.
• In patients undergoing allo-HCT, the use of rATG (regardless of dose) in combination with standard GvHD prophylaxis, compared with standard prophylaxis alone, showed no significant difference in NRM (Supplementary Figure 12) [23, 24, 25,27,29]. Moderate quality of evidence.
• In patients undergoing allo-HCT, the use of rATG in combination with standard GvHD prophylaxis, compared with standard prophylaxis alone, showed an 8% reduction in relapse risk (95% CI: 1%–15%) in the high-dose subgroup, and a 6% reduction (95% CI: 1%–10%) in the overall analysis (low- and high-risk). There was no significant difference in the low-dose subgroup (Supplementary Figure 13) [23, 24, 25,27,29]. Moderate quality of evidence.
• In patients undergoing allo-HCT, the use of rATG (regardless of dose) in combination with standard GvHD prophylaxis, compared with standard prophylaxis alone, showed no significant difference in the risk of bacterial infection (Supplementary Figure 14) and invasive fungal infection (Supplementary Figure 15) [23, 24, 25,27,28]. Low quality of evidence.
• In patients undergoing allo-HCT, the use of rATG (regardless of dose) in combination with standard GvHD prophylaxis, compared with standard prophylaxis alone, showed no significant difference in the risk of CMV reactivation (Supplementary Figure 16) [23, 24, 25,27,28]. Very Low quality of evidence.
• In patients undergoing allo-HCT, the use of rATG in combination with standard GvHD prophylaxis, compared with standard prophylaxis alone, was associated with a 2% increased risk of EBV reactivation (95% CI: 0%–4%) in the high-dose subgroup, with no significant difference observed in the overall analysis (combined low- and high-risk groups) or in the low-dose subgroup (Supplementary Figure 17) [23, 24, 25,27, 28, 29]. Very low quality of evidence.

**Recommendation**

In patients undergoing allo-HCT, the use of rATG in combination with standard GvHD prophylaxis is recommended over standard prophylaxis alone. This recommendation is based on reduced risks of aGvHD (regardless of dose), cGvHD (specifically in the high-dose subgroup), and relapse (regardless of dose).

**Quality of Evidence:** ranged from very low to moderate, depending on the outcome assessed.

**CLINICAL QUESTION 5**

What are the efficacy and risks of a CNI plus MMF regimen in patients undergoing reduced intensity and non-myeloablative conditioning allogeneic hematopoietic cell transplantation?

**Evidence Synthesis**

Quantitative data were presented as the number of events for each outcome. The absolute risks for the intervention group (CNI plus MMF) and the comparison groups (CNI alone or CNI plus methotrexate) were calculated as the ratio of the number of events to the total number of patients.

The results of the meta-analysis expressed the risk difference for the outcome between the groups, along with the 95% CI.

• There was a 16% increase (range: 7%–26%) in aGvHD-free survival with the use of CNI plus MMF compared with the control groups (CNI alone or CNI plus methotrexate). It was necessary to treat approximately seven patients to cause one additional harm (NNH: 7) (Supplementary Figure 18). Low quality of evidence [30, 31, 32, 33].
• There was an 8% increase (95% CI: 5%–11%) in aGvHD with the use of CNI plus MMF compared with the control groups (CNI alone or CNI plus methotrexate) (Supplementary Figure 19). Approximately 13 patients need to be treated to cause one additional harm (NNH: 13). Moderate quality of evidence [30, 31, 32, 33].
• There was a 12% increase (range: 8%–16%) in cGvHD risk with the use of CNI plus MMF when compared with the control groups (CNI alone or CNI plus methotrexate) (Supplementary Figure 20). It was necessary to treat approximately nine patients to cause one additional harm (NNH: 9). Moderate quality of evidence [30, 31, 32, 33].
• There was a 17% reduction (range: 6%–29%) in mortality risk with the use of CNI plus MMF when compared to the control groups (CNI alone or CNI plus methotrexate) (Supplementary Figure 21). It was necessary to treat approximately six patients to prevent one additional death (NNT: 6). Low quality of evidence [30, 31, 32, 33].
• There was no significant difference in the relapse or progression with the use of CNI plus MMF compared with the control groups (CNI alone or CNI plus methotrexate) (Supplementary Figure 22). Very Low quality of evidence [30, 31, 32, 33].
• There was a 3% increase (95% CI: 1%–6%) in the probability of GRFS with the use of CNI plus MMF compared with the control group (CNI alone or CNI plus methotrexate) (Supplementary Figure 23). It was necessary to treat 33 patients to achieve one additional favorable GRFS outcome (NNT: 33). Moderate quality of evidence [30, 31, 32].

**Recommendation**

In patients undergoing reduced intensity and non-myeloablative conditioning allo-HCT, the use of CNI plus MMF compared with a CNI alone or CNI plus methotrexate is associated with the following outcomes: an increase in the probability of GRFS and an increased risk of both aGvHD and cGvHD. However, the CNI plus MMF regimen can be recommended because it is associated with a reduction in death risk, and no difference in the relapse or progression, despite the higher risks of aGvHD and cGvHD.

**Quality of evidence:** varied between very low and moderate, depending on the analyzed outcome.

**CLINICAL QUESTION 6**

What are the efficacy and risks of a regimen with high-dose post-transplant cyclophosphamide (PTCy) regimen (50 mg/kg on Day +3 and Day +4) plus a CNI with or without MMF in patients undergoing haploidentical allogeneic hematopoietic cell transplantation for malignant diseases, using either myeloablative or non-myeloablative conditioning and bone marrow or peripheral blood grafts?

**Evidence Synthesis**

In patients undergoing haploidentical allo-HCT for malignant diseases, using either myeloablative or non-myeloablative conditioning and bone marrow or peripheral blood grafts, the regimen with high-dose PTCy plus CNI with or without MMF produced the following effects, when compared with high-dose PTCy plus sirolimus and MMF [34, 35, 36]:

• There was no significant difference in Grade II-IV aGvHD risk (Supplementary Figure 24).
• There was no significant difference in Grade III-IV aGvHD risk (Supplementary Figure 25).
• There was no significant difference in cGvHD risk (Supplementary Figure 26).
• There was no significant difference in OS (Supplementary Figure 27).
• There was no significant difference in EFS (Supplementary Figure 28).
• There was no significant difference in NRM risk (Supplementary Figure 29).
• There was no significant difference in CMV reactivation (Supplementary Figure 30).
• There was a 21% reduction (95% CI: 12%−31%) in hemorrhagic cystitis risk with the use of high-dose PTCy plus CNI with or without MMF compared with high-dose PTCy plus sirolimus and MMF (Supplementary Figure 31).
• There was a 11% reduction (95% CI: 5%−17%) of sinusoidal obstructive syndrome risk with the use high-dose PTCy plus a CNI with or without MMF compared with high-dose PTCy plus sirolimus and MMF (Supplementary Figure 32).

**Recommendations**

In patients undergoing haploidentical allo-HCT for malignant diseases, using either myeloablative or non-myeloablative conditioning and bone marrow or peripheral blood grafts, a regimen with high-dose PTCy plus CNI with or without MMF, compared with high-dose PTCy plus sirolimus and MMF, reduces hemorrhagic cystitis and sinusoidal obstructive syndrome risks, despite showing no significant differences in the risk of Grade II–IV or III–IV aGvHD, cGvHD, NRM, OS, or EFS.

**CLINICAL QUESTIONS 7a and 7b**

**7a.** What are the efficacy and risks of regimens involving high-dose PTCy alone, or high-dose PTCy plus a CNI (with or without MMF), in patients undergoing allo-HCT with matched related or unrelated donors?

**Evidence Synthesis**

In patients undergoing allo-HCT with related or unrelated matched donors, regimens utilizing high-dose PTCy alone or high-dose PTCy plus a CNI (with or without MMF) demonstrated the following effects when compared with regimens without high-dose PTCy:

Randomized controlled trials [12,37,38]

• There was no significant difference in Grade II-IV aGvHD risk (Supplementary Figure 33).
• There was no significant difference in Grade III-IV aGvHD risk (Supplementary Figure 34).
• There was no significant difference in OS (Supplementary Figure 35).
• There was no significant difference in EFS (Supplementary Figure 36).
• There was no significant difference in NRM risk (Supplementary Figure 37).
• There was no significant difference in CMV reactivation (Supplementary Figure 38).
• There was no significant difference in EBV reactivation (Supplementary Figure 39).
• There was an 11% reduction (95% CI: 3%–18%) in cGvHD risk using regimens involving high-dose PTCy compared with regimens without high-dose PTCy in randomized controlled trials (Supplementary Figure 40).

Observational Cohorts [39, 40, 41, 42, 43, 44]

• There was a 22% reduction (95% CI: 12%–31%) in the risk of Grade II–IV aGvHD when using PTCy regimens, compared with regimens without PTCy (Supplementary Figure 41).
• In cohorts, there was a 18% reduction (95% CI: 5%–31%) in the risk of aGvHD Grade III-IV, when using PTCy regimens compared with regimens without PTCy (Supplementary Figure 42).
• In cohorts, there was a 18% (95% CI: 12%–24%) reduction in the risk of cGvHD when using PTCy regimens compared with regimens without PTCy (Supplementary Figure 43).
• In cohorts, there was a 13% (95% CI: 6%–19%) increase in OS rate when using PTCy regimens compared with regimens without PTCy (Supplementary Figure 44).
• In cohorts, there was an 8% (95% CI: 0%–16%) increase in EFS Rate when using PTCy regimens compared with regimens without PTCy (Supplementary Figure 45).
• In cohorts, there was a 5% (95% CI: 1%–9%) reduction in NRM when using PTCy regimens compared with regimens without PTCy (Supplementary Figure 46).
• In cohorts, there was a 23% (95% CI: 11%–35%) increase in GRFS rate when using PTCy regimens compared with regimens without PTCy (Supplementary Figure 47).
• In cohorts, there was a 16% (95% CI: 8%–25%) increase in hemorrhagic cystitis risk when using PTCy regimens compared with regimens without PTCy (Supplementary Figure 48).
• There was no significant difference in sinusoidal obstructive syndrome (Supplementary Figure 49).
• There was no significant difference in CMV reactivation (Supplementary Figure 50).

In cohorts, for patients undergoing allo-HCT with related and unrelated matched donors, the regimen with a high-dose PTCy plus CNI, compared with a high-dose PTCy plus CNI plus MMF, produced the following effects [45,46]:

• There was no significant difference in the risk of Grade II–IV aGvHD (Supplementary Figure 51).
• There was no significant difference in the risk of Grade III–IV aGvHD (Supplementary Figure 52).
• There was no significant difference in the risk of cGvHD (Supplementary Figure 53).
• There was no significant difference in the OS rate (Supplementary Figure 54).
• There was no significant difference in the EFS (Supplementary Figure 55).
• There was no significant difference in NRM risk (Supplementary Figure 56).

**Recommendation**

In patients undergoing allo-HCT with related and unrelated matched donors, the use of high-dose PTCy, either alone or combined with a CNI (with or without MMF), compared with regimens without high-doses of PTCy is associated with reduced risks of cGvHD, Grade II–IV aGvHD, Grade III–IV aGvHD, and NRM compared with regimens that do not include high-dose PTCy. In addition, PTCy- based regimens improve OS, EFS, GRFS, and increase the risk of hemorrhagic cystitis.

In cohorts, for patients undergoing allo-HCT with related and unrelated matched donors, the regimen with a high-dose PTCy plus CNI, compared with CNI plus MMF, shows no significant difference in Grade II–IV aGvHD, Grade III-IV aGvHD, cGvHD, NRM, OS, and EFS.

**7b** What are the efficacy and risks of a high-dose PTCy plus CNI and MMF or sirolimus, compared with regimens without PTCy, in patients undergoing allogeneic hematopoietic cell transplantation with unrelated mismatched donors?

**Evidence Synthesis**

In unrelated mismatched donors a regimen with high-dose PTCy plus a CNI and MMF or sirolimus, compared with regimens without PTCy produced the following effects:

Randomized controlled trials [9,47]:

• There was no significant difference in Grade II-IV aGvHD risk (Supplementary Figure 57).
• There was no significant difference in OS (Supplementary Figure 58).
• There was a 10% reduction (95% CI: 6%–14%) in Grade III-IV aGvHD risk when using regimens with PTCy compared with regimens without PTCy (Supplementary Figure 59).
• There was a 22% reduction (95% CI: 4%–40%) in cGvHD risk, when using PTCy-based regimens compared with regimens without PTCy (Supplementary Figure 60).

Observational Cohorts [48, 49, 50, 51]

• There was no significant difference in Grade II-IV aGvHD risk (Supplementary Figure 61).
• There was no significant difference in Grade III-IV aGvHD risk (Supplementary Figure 62).
• There was no significant difference in cGvHD risk (Supplementary Figure 63).
• There was a 23% increase (95% CI: 14%–33%) in OS when using regimens with PTCy compared with regimens without PTCy (Supplementary Figure 64).
• There was no significant difference in EFS (Supplementary Figure 65).
• There was no significant difference in NRM (Supplementary Figure 66).
• In cohorts, there was a 9% reduction (95% CI: 1%–18%) in hemorrhagic cystitis, when using PTCy-based regimens compared with regimens without PTCy (Supplementary Figure 67).
• In cohorts, there was a 25% reduction (95% CI: 2%–47%) in CMV reactivation risk, using PTCy regimens, compared with regimens without PTCy (Supplementary Figure 68).

**Recommendation**

In unrelated mismatched donor transplants, regimens utilizing high-dose PTCy plus a CNI and MMF (or sirolimus) are associated with a reduced risk of Grade III–IV aGvHD, cGvHD, hemorrhagic cystitis, and CMV reactivation compared to regimens without PTCy. Furthermore, these regimens are associated with improved OS, with no significant differences observed in Grade II–IV aGvHD, NRM, or EFS.

**CLINICAL QUESTION 8**

What are the efficacy and risks of the regimen with high-dose PTCy plus ATG in patients undergoing allogeneic hematopoietic cell transplantation with haploidentical, related or unrelated matched donors or unrelated mismatched donors?

**Evidence Synthesis**

In patients undergoing allo-HCT from haploidentical, related or unrelated matched donors or unrelated mismatched donors, the addition of high-dose PTCy plus ATG, compared with regimens lacking this combination, produces the following effects [40,52, 53, 54, 55, 56, 57, 58, 59]:

• There was a reduction in the risk of Grade II–IV aGvHD among patients treated with PTCy plus ATG compared with regimens that did not include this combination. The estimated risks were 9% (95% CI: 8%–16%) in the overall analysis (combining the haploidentical and non-haploidentical subgroups) and 13% (95% CI: 5%–21%) in the haploidentical subgroup. This effect was not observed in the non-haploidentical subgroup when analyzed separately (Supplementary Figure 69).
• There was no significant difference in the overall risk of Grade III–IV aGvHD between patients treated with PTCy plus ATG and those receiving regimens without this combination, regardless of donor type (haploidentical or non-haploidentical) (Supplementary Figure 70).
• There was a reduction in the risk of cGvHD among patients treated with PTCy plus ATG compared with regimens that did not include this combination. The estimated risks were 9% (95% CI: 1%–17%) in the overall analysis (combining haploidentical and non-haploidentical subgroups). This effect was not observed in the haploidentical and non-haploidentical subgroups when analyzed separately (Supplementary Figure 71).
• There was no significant difference in the aggregate OS rate between patients treated with PTCy plus ATG and those treated with other regimens, regardless of donor type (haploidentical or non-haploidentical) (Supplementary Figure 72).
• There was no significant difference in the aggregate EFS in patients treated with PTCy plus ATG compared with other regimens, regardless of donor type (haploidentical or non-haploidentical) (Supplementary Figure 73).
• There was no significant difference in the aggregate NRM risk in patients treated with PTCy plus ATG compared with other regimens, regardless of donor type (haploidentical or non-haploidentical) (Supplementary Figure 74).
• There was no significant difference in the aggregate GRFS rate in patients treated with PTCy plus ATG compared with other regimens without this combination, regardless of donor type (haploidentical or non-haploidentical) (Supplementary Figure 75).
• There was no significant difference in the aggregate CMV reactivation risk in patients treated with PTCy plus ATG compared with other regimens, regardless of donor type (haploidentical or non-haploidentical) (Supplementary Figure 76).
• There was no significant difference in the aggregate hemorrhagic cystitis risk in patients treated with PTCy plus ATG compared with other regimens, regardless of donor type (haploidentical or non-haploidentical) (Supplementary Figure 77).
• There was an increase in the EBV reactivation risk in patients treated with PTCy plus ATG compared with other regimes. The estimated risks were 24% (95% CI: 10%–37%), 42% (95% CI: 23%–61%), and 18% (95% CI: 10%–26%), in the overall analysis (combining haploidentical and non-haploidentical subgroups), the haploidentical subgroup, and the -non-haploidentical subgroup, respectively (Supplementary Figure 78).

**Recommendations**

In patients undergoing allo-HCT with haploidentical, related or unrelated matched donors or unrelated mismatched donors, the regimen combining high-dose PTCy plus ATG is associated with reduced risks of Grade II-IV aGvHD, and cGvHD, as well an increased risk of EBV reactivation when compared with other regimens lacking this combination. Despite these differences, the two regimens show no significant difference in terms of Grade III-IV aGvHD, OS, EFS, NRM, GRFS, CMV reactivation and hemorrhagic cystitis.

**CLINICAL QUESTION 9**

What are the efficacy and risks of the regimen with tacrolimus plus sirolimus in patients undergoing myeloablative allogeneic hematopoietic cell transplantation with related or unrelated matched donors?

**Evidence Synthesis**

It was possible to pool the outcomes (aGvHD, cGvHD, and mortality) from three randomized clinical trials included in this analysis [20,21,60].

• There was no significant difference in the risk of aGvHD when comparing the sirolimus plus tacrolimus prophylaxis regimen with tacrolimus plus methotrexate or cyclosporine plus methotrexate.
• There was no significant difference in the risk of cGvHD between the sirolimus plus tacrolimus regimen and the tacrolimus plus methotrexate or cyclosporine plus methotrexate regimens.
• Mortality rates were also similar across the groups, with no significant difference observed between sirolimus plus tacrolimus and either comparator regimen.

**Recommendations**

In patients undergoing myeloablative allo-HCT with related or unrelated matched donors, GvHD prophylaxis with sirolimus plus tacrolimus does not demonstrate greater benefit than tacrolimus plus methotrexate or cyclosporine plus methotrexate in terms of aGvHD, cGvHD, or mortality.

**CLINICAL QUESTION 10**

What are the efficacy and risks of a triple-drug prophylaxis regimen consisting of a CNI, MMF, and sirolimus in patients undergoing reduced-intensity or non-myeloablative allogeneic hematopoietic cell transplantation from related or unrelated (matched or mismatched) donors?

**Evidence Synthesis**

• In patients undergoing non-myeloablative conditioning allo-HCT with an unrelated matched donor, replacing tacrolimus plus MMF with a combination of cyclosporine, MMF, and sirolimus resulted in a lower incidence of Grade II–IV aGvHD and improved two-year OS [61]. Low quality of evidence.
• For GvHD prophylaxis in patients undergoing non-myeloablative conditioning allo-HCT with a related or unrelated mismatched donor, the addition of sirolimus to cyclosporine and MMF resulted in a lower incidence of aGvHD and superior OS [62]. Very Low quality of evidence.
• In patients undergoing non-myeloablative conditioning allo-HCT with an unrelated matched or mismatched donor, adding sirolimus to cyclosporine and MMF resulted in a significantly lower incidence of acute GvHD [63]. Low quality of evidence.

**Recommendations**

In patients undergoing reduced intensity or non-myeloablative allo-HCT with related or unrelated matched or mismatched donors, cyclosporine plus MMF and sirolimus led to a reduction in the incidence of Grade II-IV aGvHD and an improved two-year OS.

**Quality of evidence**: varied between very low and low.

**CLINICAL QUESTION 11**

What is the efficacy and risks of the abatacept plus a CNI and methotrexate regimen in patients undergoing non-myeloablative or myeloablative allogeneic hematopoietic cell transplantation with an unrelated matched or mismatched donors?

**Evidence Synthesis**

In patients undergoing non-myeloablative or myeloablative allo-HCT with an unrelated matched or mismatched donor, the abatacept plus CNI and methotrexate regimen produced the following effects:

In randomized clinical trials, the use of this regimen was associated with a lower risk of Grade III–IV aGvHD (28% risk reduction) at three months of follow-up and a lower risk of relapse (12% risk reduction) at 24 months of follow-up, with no significant difference in the cGvHD risk and OS compared with a methotrexate plus CNI regimen [64,65].

**Recommendations**

In patients undergoing non-myeloablative or myeloablative allo-HCT with matched and mismatched unrelated donors, bone marrow or peripheral blood, the abatacept plus CNI and methotrexate regimen produces lower risk of aGvHD III-IV and relapse, with no significant difference in the risk of cGvHD and OS, when compared to methotrexate combined with a CNI.

**Quality of evidence:** very low

## Conclusions

Preferred approaches to GvHD prophylaxis vary according to transplant characteristics, including donor–recipient matching, conditioning intensity, graft source, and other clinical factors. The choice of GvHD prophylaxis should therefore be guided by donor type (matched sibling, matched unrelated, or haploidentical), conditioning regimen (myeloablative versus non-myeloablative/reduced intensity), and graft source (peripheral blood or bone marrow).

This systematic review of GvHD prophylaxis addressed 11 clinical questions encompassing patients who underwent allo-HCT using myeloablative or non-myeloablative/reduced-intensity conditioning regimens with haploidentical, matched sibling, matched unrelated, or mismatched unrelated donors.

### Choosing a graft-versus-host disease prophylaxis regimen

*1. Calcineurin Inhibitor plus methotrexate in myeloablative regimens:*

For allo-HCT using matched related or matched unrelated donors, cyclosporine, or tacrolimus in combination with methotrexate reduces the risk of acute and chronic GvHD without affecting mortality.

*2. Calcineurin inhibitor plus mycophenolate mofetil in myeloablative regimens:*

For allo-HCT using matched related or matched unrelated donors, regimens using cyclosporine or tacrolimus plus MMF do not differ from methotrexate-based combinations in terms of aGvHD, cGvHD, mortality, relapse, or infection rates. However, MMF-based regimens can be recommended due to lower toxicity (nephrotoxicity and respiratory failure, particularly when combined with tacrolimus) and the lower incidence of mucositis (regardless of whether cyclosporine or tacrolimus is used).

*3. Anti-thymocyte Globulin (rATG) <6* *mg/kg (low dose) or ≥6* *mg/kg (high dose) in peripheral blood sibling and unrelated donors:*

The use of rATG in combination with standard GvHD prophylaxis is recommended over standard prophylaxis alone, as it reduces the risk of aGvHD and relapse (regardless of dose), as well as cGvHD (specifically in the low-dose subgroup).

*4. A calcineurin inhibitor plus mycophenolate mofetil in non-myeloablative/reduced intensity conditioning regimens:*

The use of a CNI plus MMF compared with a CNI alone or a CNI plus methotrexate is associated with an increase in the probability of GRFS and an increased risk of both aGvHD and cGvHD. The CNI plus MMF regimen can be recommended because it is associated with a reduction in death risk, and no difference in the relapse or progression, despite the higher risks of aGvHD and cGvHD.

*5. Cyclophosphamide*

*a. Nonmyeloablative and myeloablative haploidentical allo-HCT*

A high-dose PTCy plus CNI regimen (with or without MMF) reduces the risks of hemorrhagic cystitis and sinusoidal obstructive syndrome compared with high-dose PTCy plus sirolimus and MMF, despite having no significant differences in the risk of Grade II–IV or III–IV aGvHD, cGvHD, NRM, OS and EFS.

*b. Related and unrelated matched donors*

A high-dose PTCy regimen, used alone or combined with a CNI (with or without MMF), is associated with reduced risks of cGvHD, Grade II–IV aGvHD, Grade III–IV aGvHD, and NRM, compared with regimens that do not include high-dose PTCy. In addition, PTCy-based regimens improve OS, DFS, and GRFS, but increase hemorrhagic cystitis risk. In cohort studies, for patients undergoing allo-HCT with related and unrelated matched donors, the regimen with a high-dose PTCy plus CNI, compared with CNI plus MMF, shows no significant difference in Grade II–IV aGvHD, Grade III-IV aGvHD, cGvHD, NRM, OS, and EFS.

*c. Unrelated mismatched donors*

A high-dose PTCy plus CNI regimen (combined with either MMF or sirolimus) is associated with reduced risks of Grade III–IV aGvHD, cGvHD, hemorrhagic cystitis, and CMV reactivation compared with regimens without PTCy. Furthermore, this approach yielded an OS benefit, notwithstanding the lack of significant differences in Grade II–IV aGvHD, NRM, or EFS.

*6. Cyclophosphamide plus antithymocyte globulin (ATG)*

High-dose PTCy plus ATG for related or unrelated matched donors or unrelated mismatched donors is associated with reduced risks of Grade II-IV aGvHD, and cGvHD, as well as an increased risk of EBV reactivation when compared with other regimens lacking this combination. Despite these differences, the two regimens show no significant differences in outcomes such as Grade III-IV aGvHD, OS, EFS, NRM, GRFS, CMV reactivation and hemorrhagic cystitis.

7. *Tacrolimus plus sirolimus in myeloablative conditioning regimens*

The use of sirolimus plus tacrolimus for related or unrelated matched donors does not demonstrate greater benefit than tacrolimus plus methotrexate or cyclosporine plus methotrexate in terms of aGvHD, cGvHD, and mortality.

8. *Calcineurin inhibitor plus mycophenolate mofetil and sirolimus in non-myeloablative / reduced intensity conditioning*

The use of cyclosporine plus MMF and sirolimus for related or unrelated matched or mismatched donors leads to a reduction in the incidence of Grade II-IV aGvHD and improved two-year OS.

9. *Abatacept plus calcineurin inhibitor and methotrexate*

For patients undergoing myeloablative or non-myeloablative allo-HCT (using bone marrow or peripheral blood from matched or mismatched unrelated donors), the addition of abatacept to a CNI and methotrexate regimen is associated with a lower risk of Grade III–IV aGvHD and relapse, with no significant difference in the risk of cGvHD or OS, compared to a CNI plus methotrexate alone.

This systematic review of GvHD prophylaxis did not address questions regarding the optimal prophylactic regimen for specific clinical contexts such as myeloablative versus reduced-intensity or non-myeloablative conditioning, related versus unrelated donors, or bone marrow versus peripheral blood graft sources but it represents an important step toward defining preferred GvHD prophylaxis strategies. In addition, despite limited long-term data, abatacept represents a promising strategy, particularly in unrelated donor transplantation. Furthermore, PTCy has emerged as a novel approach to GvHD prophylaxis beyond the haploidentical transplantation setting.

These guidelines are limited by the predominance of observational studies, heterogeneity of conditioning regimens, and evolving transplant platforms. However, these recommendations are particularly relevant for low- and middle-income countries, where access to novel agents and graft manipulation remains limited.

## Data availability

The data that support the findings of this study are available from the corresponding author upon reasonable request.

## Appendix Supplementary materials

Image, application 1

## Footnotes

Supplementary material associated with this article can be found in the online version at doi:10.1016/j.htct.2026.106494.
