# Supplementary material for: Prophylaxis of graft-versus-host-disease: Systematic evidence-based recommendations of the Brazilian association of hematology, hemotherapy, and cell therapy (ABHH) and the Brazilian society of cellular therapy and bone marrow transplantation (SBTMO)
Source: Hematol Transfus Cell Ther. 2026 Jul 9;48(3):106494. doi: 10.1016/j.htct.2026.106494 (PMC13382006; doi:10.1016/j.htct.2026.106494)
Supplement: Supplementary file 1 [file mmc1.pdf]

Prophylaxis of graft-versus-host-disease: Systematic Evidence-Based Recommendations of the Brazilian Association of Hematology, Hemotherapy, and Cell Therapy (ABHH) and the Brazilian Society of Cellular Therapy and Bone Marrow Transplantation (SBTMO)

Supplementary File

Meta-analysis Figures (Forests plots)

Supplementary Material: Master Abbreviation Key

The following abbreviations are used uniformly throughout Supplementary Figures 1 to 78:

- **aGvHD: Acute graft-versus-host disease**
- **ATG: Anti-thymocyte globulin**
- **cGvHD: Chronic graft-versus-host disease**
- **CNI: Calcineurin inhibitor (e.g., cyclosporine, tacrolimus)**
- **CMVR: Cytomegalovirus reactivation**
- **EBVR: Epstein-Barr virus reactivation**
- **EFS: Event-free survival**
- **GRFS: GvHD-free, relapse-free survival**
- **HC: Hemorrhagic cystitis**
- **MMF: Mycophenolate mofetil**
- **MTX: Methotrexate**
- **NRM: Non-relapse mortality**
- **OS: Overall survival**
- **PTCy: Post-transplant cyclophosphamide**
- **SOS: Sinusoidal obstruction syndrome**

CLINICAL QUESTION 3

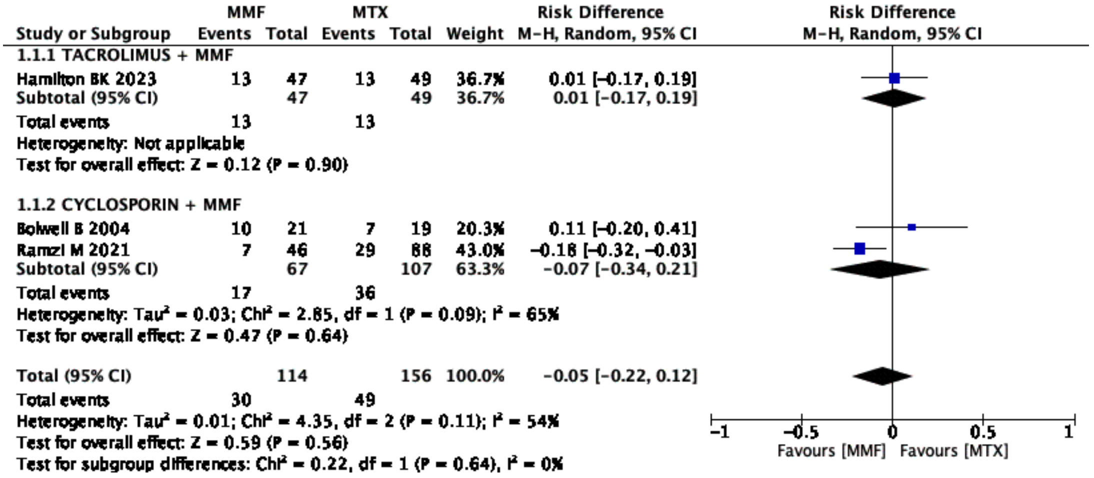

Figure 1: aGVHD analysis: comparing the combination of CNI plus MMF versus the combination with MTX, regardless of the use of tacrolimus or cyclosporine

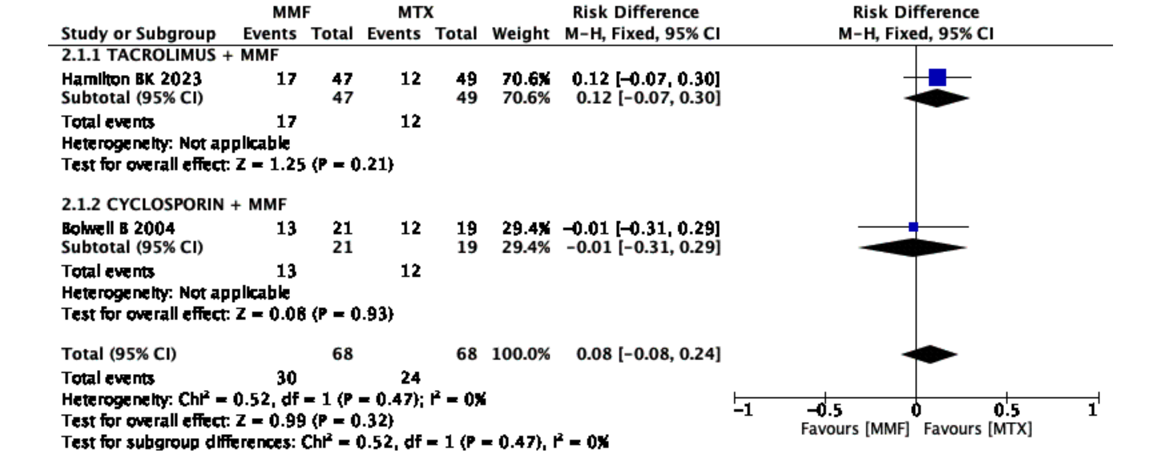

Figure 2: cGVHD analysis: comparing the combination of CNI plus MMF versus the combination with MTX, regardless of the use of tacrolimus or cyclosporine

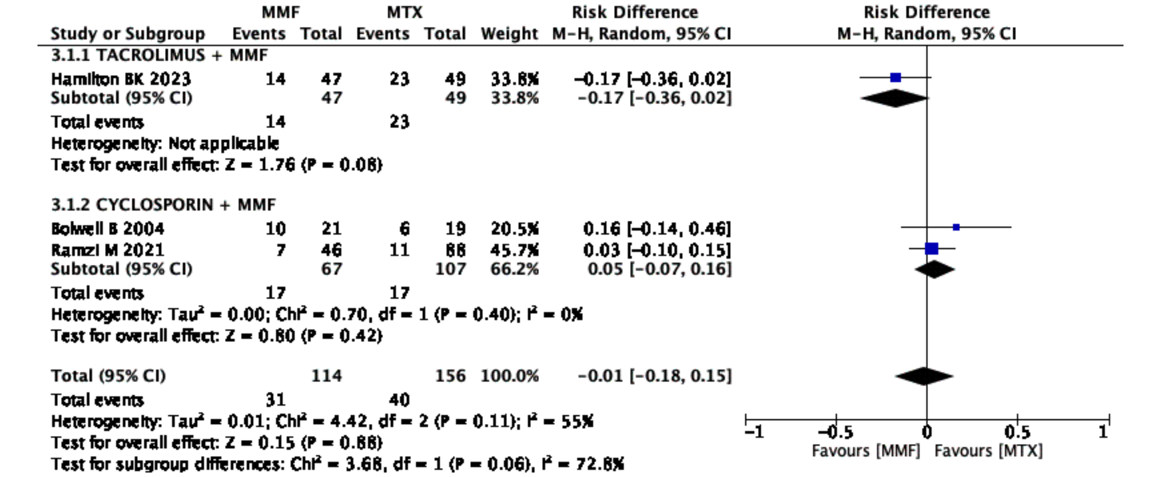

Figure 3: Mortality analysis: comparing the combination of CNI plus MMF versus the combination with MTX, regardless of the use of tacrolimus or cyclosporine

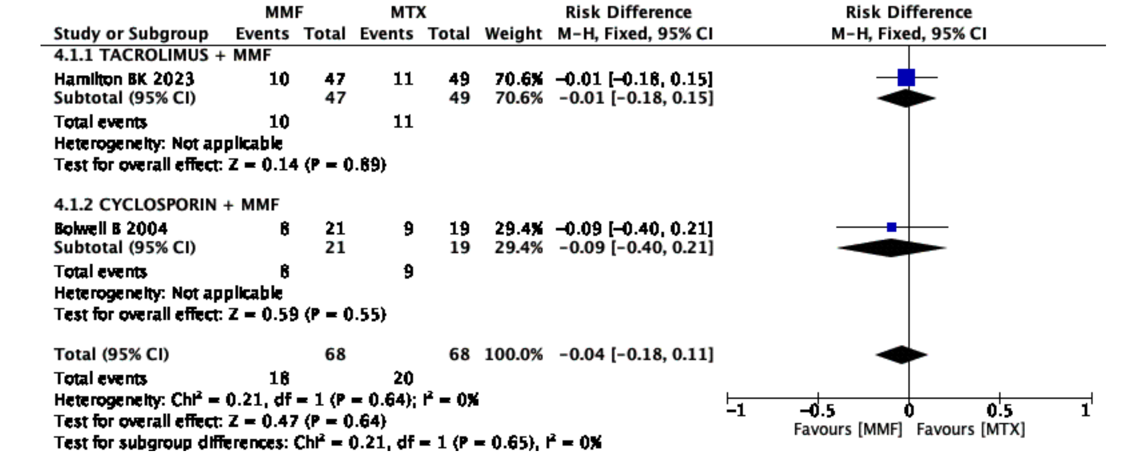

Figure 4: Relapse analysis: comparing the combination of CNI plus MMF versus the combination with MTX, regardless of the use of tacrolimus or cyclosporine

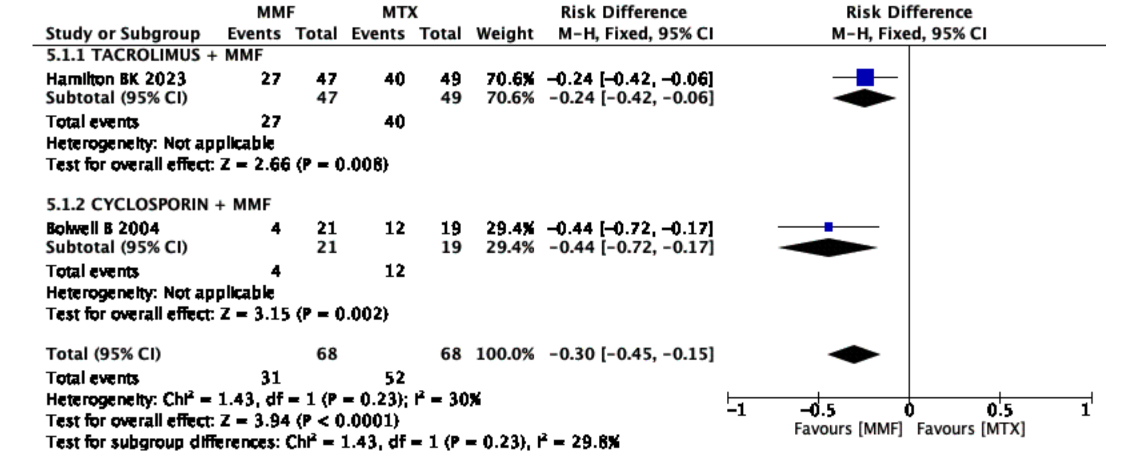

Figure 5: Mucositis analysis: comparing the combination of CNI plus MMF versus the combination with MTX, regardless of the use of tacrolimus or cyclosporine

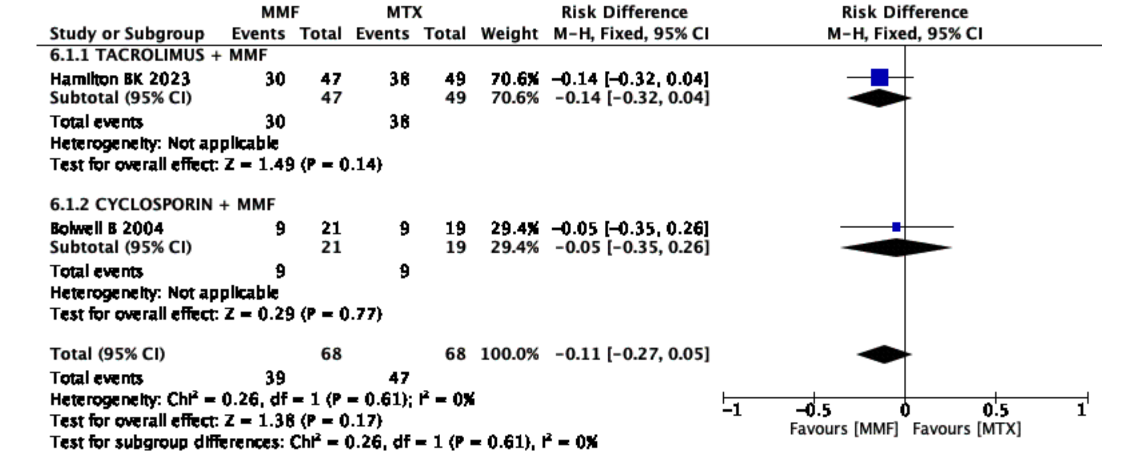

Figure 6: Infection analysis: comparing the combination of CNI plus MMF versus the combination with MTX, regardless of the use of tacrolimus or cyclosporine

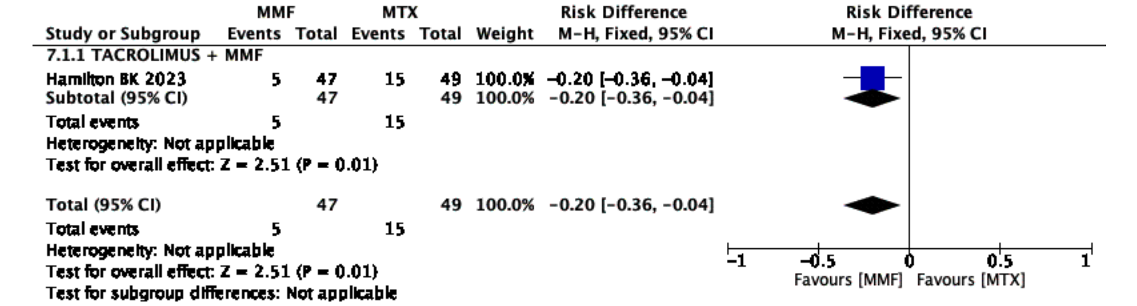

Figure 7: Toxicity analysis: comparing the combination of CNI plus MMF versus the combination with MTX, regardless of the use of tacrolimus or cyclosporine

CLINICAL QUESTION 4

Study or Subgroup	ATG Events	ATG Total	no ATG Events	no ATG Total	Weight	Risk Difference M-H, Fixed, 95% CI
**1.1.1 LOW DOSE (< 6mg/kg)**						
Chang YJ2020	18	132	35	131	20.1%	−0.13 [−0.23, −0.04]
Cho BS2021	14	60	19	60	9.2%	−0.08 [−0.24, 0.08]
Walker I 2016	50	101	66	102	15.5%	−0.15 [−0.29, −0.02]
Subtotal (95% CI)		293		293	44.7%	−0.13 [−0.20, −0.06]
Total events	82		120			
Heterogeneity: Chi² = 0.43, df = 2 (P = 0.81); I² = 0%						
Test for overall effect: Z = 3.53 (P = 0.0004)						
**1.1.2 HIGH DOSE (≥6 mg/kg)**						
Bacigalupo A 2001	20	56	40	53	8.3%	−0.40 [−0.57, −0.23]
Finke J 2009	34	103	50	99	15.4%	−0.17 [−0.31, −0.04]
Kröger N 2016	9	83	13	72	11.8%	−0.07 [−0.18, 0.04]
Soiffer RJ2017	23	128	40	132	19.8%	−0.12 [−0.23, −0.02]
Subtotal (95% CI)		370		356	55.3%	−0.17 [−0.23, −0.11]
Total events	86		143			
Heterogeneity: Chi² = 10.53, df = 3 (P = 0.01); I² = 72%						
Test for overall effect: Z = 5.23 (P < 0.00001)						
**Total (95% CI)**		663		649	100.0%	−0.15 [−0.20, −0.10]
Total events	168		263			
Heterogeneity: Chi² = 11.19, df = 6 (P = 0.08); I² = 46%						
Test for overall effect: Z = 6.24 (P < 0.00001)						
Test for subgroup differences: Chi² = 0.67, df = 1 (P = 0.41), I² = 0%						

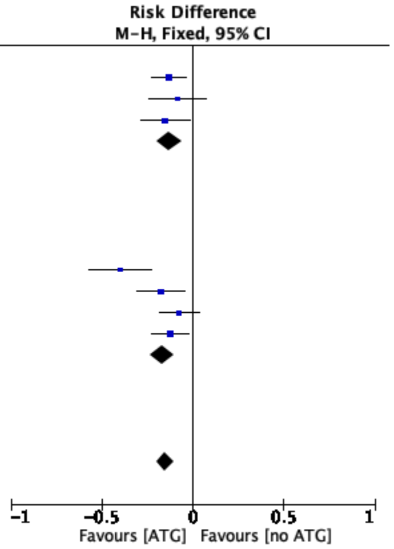

Figure 8: Analysis of the risk of aGVHD and subgroup by dose - ATG (low or high dose) vs no ATG

Study or Subgroup	ATG Events	ATG Total	no ATG Events	no ATG Total	Weight	Risk Difference M-H, Fixed, 95% CI
**1.2.1 LOW DOSE (< 6mg/kg)**						
Chang YJ2020	37	132	69	131	20.1%	−0.25 [−0.36, −0.13]
Cho BS2021	27	60	9	60	9.2%	0.30 [0.15, 0.45]
Walker I 2016	22	101	33	102	15.5%	−0.11 [−0.23, 0.02]
Subtotal (95% CI)		293		293	44.7%	−0.09 [−0.16, −0.01]
Total events	86		111			
Heterogeneity: Chi² = 31.44, df = 2 (P < 0.00001); I² = 94%						
Test for overall effect: Z = 2.28 (P = 0.02)						
**1.2.2 HIGH DOSE (≥6 mg/kg)**						
Bacigalupo A 2001	22	56	32	53	8.3%	−0.21 [−0.39, −0.03]
Finke J 2009	12	103	42	99	15.4%	−0.31 [−0.42, −0.19]
Kröger N 2016	26	83	49	72	11.8%	−0.37 [−0.51, −0.22]
Soiffer RJ2017	16	128	38	132	19.8%	−0.16 [−0.26, −0.07]
Subtotal (95% CI)		370		356	55.3%	−0.25 [−0.32, −0.19]
Total events	76		161			
Heterogeneity: Chi² = 6.78, df = 3 (P = 0.08); I² = 56%						
Test for overall effect: Z = 7.91 (P < 0.00001)						
**Total (95% CI)**		663		649	100.0%	−0.18 [−0.23, −0.13]
Total events	162		272			
Heterogeneity: Chi² = 50.76, df = 6 (P < 0.00001); I² = 88%						
Test for overall effect: Z = 7.31 (P < 0.00001)						
Test for subgroup differences: Chi² = 11.57, df = 1 (P = 0.0007), I² = 91.4%						

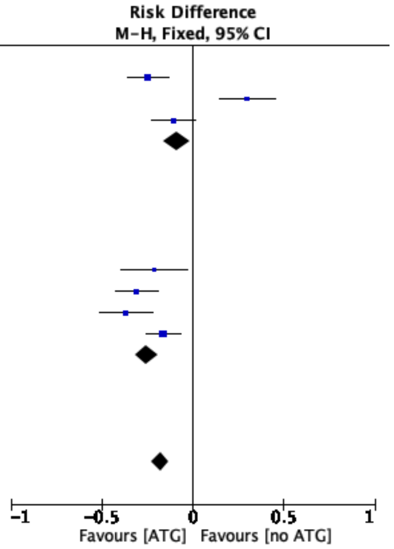

Figure 9: Analysis of the risk of cGVHD and subgroup by dose - ATG (low or high dose) vs no ATG

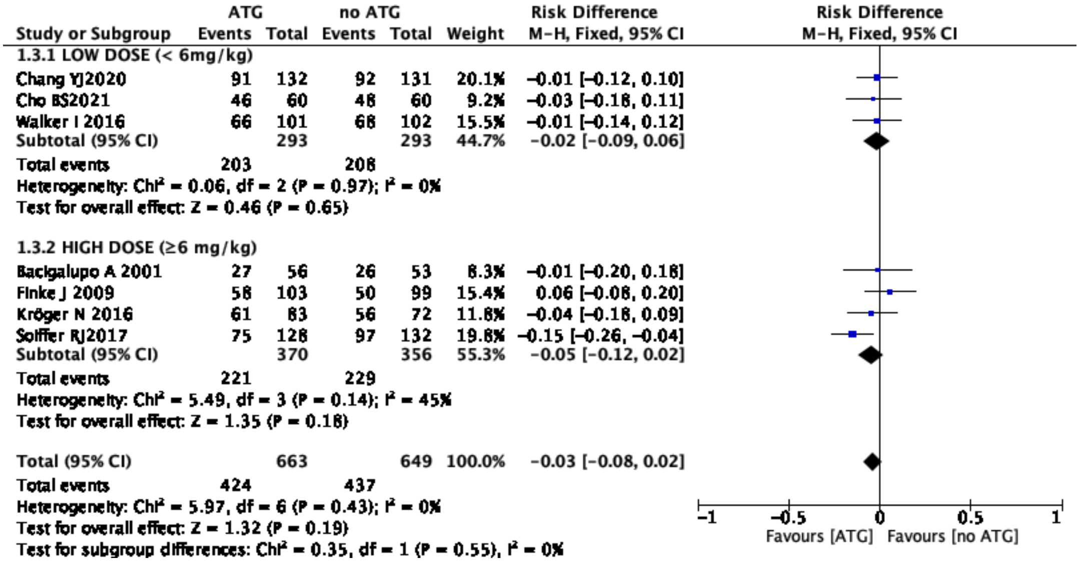

Figure 10: Analysis of OS and subgroup by dose - ATG (low or high dose) vs no ATG

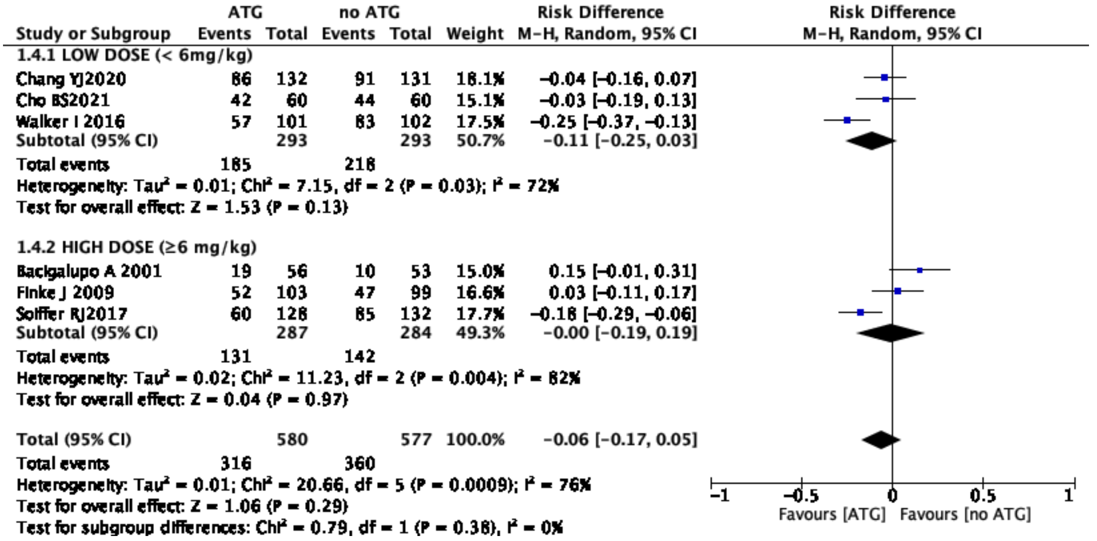

Figure 11: Analysis of the risk of disease-free survival and subgroup by dose - ATG (low or high dose) vs no ATG

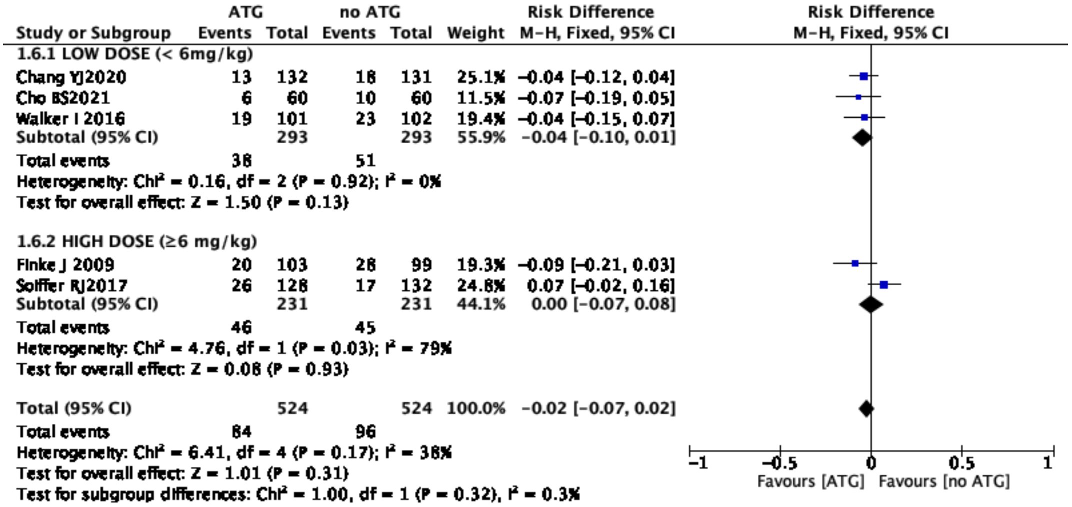

Figure 12: Analysis of the risk of NRM and subgroup by dose - ATG (low or high dose) vs no ATG

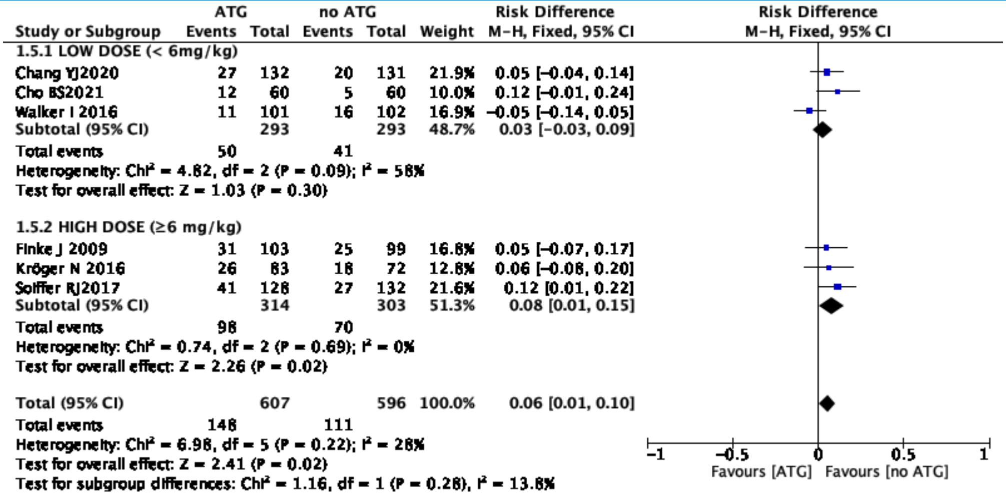

Figure 13: Analysis of the risk of relapse and subgroup by dose - ATG (low or high dose) vs no ATG

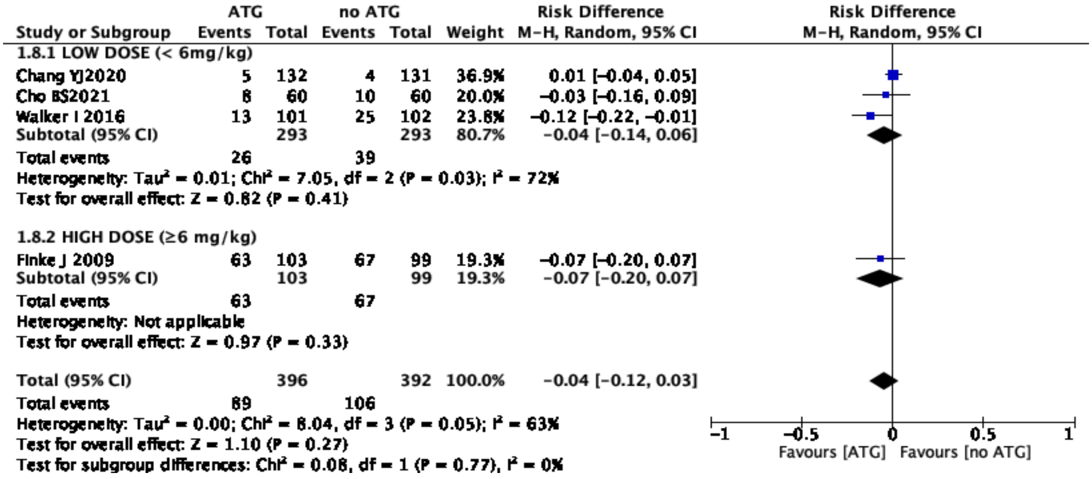

Figure 14: Analysis of the risk of bacterial infection and subgroup by dose - ATG (low or high dose) vs no ATG

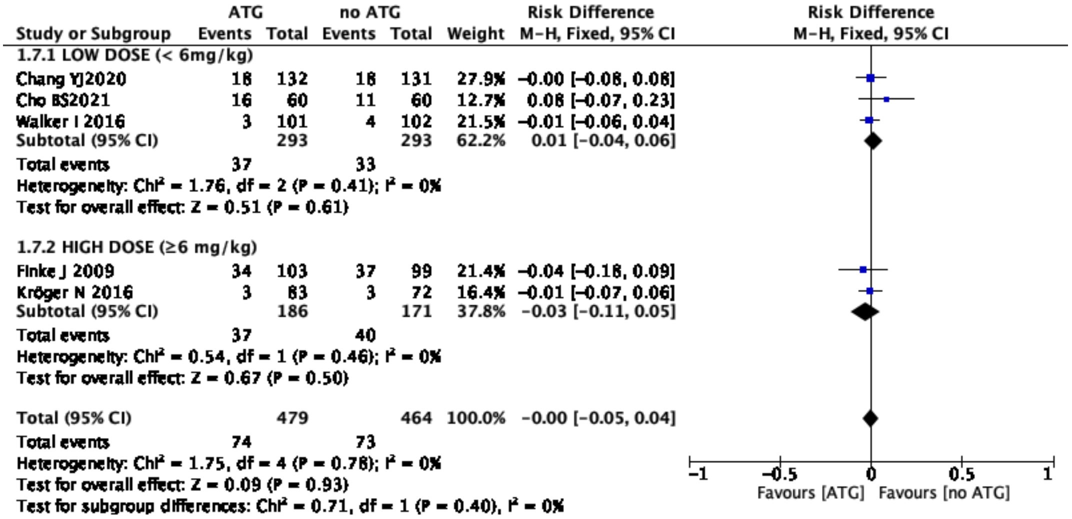

Figure 15: Analysis of the risk of invasive fungal infection and subgroup by dose - ATG (low or high dose) vs no ATG

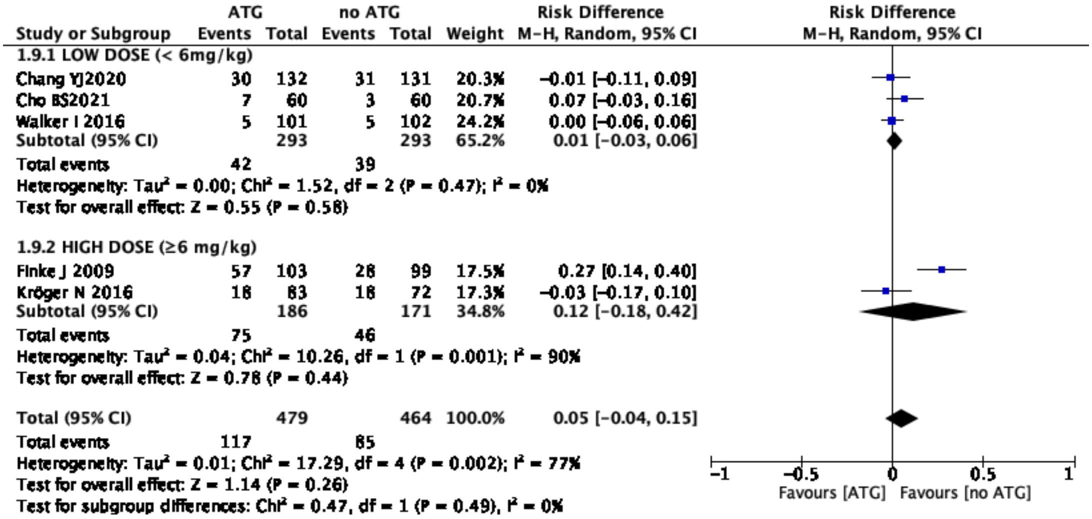

Figure 16: Analysis of the risk of CMVR and subgroup by dose - ATG (low or high dose) vs no ATG

Study or Subgroup	ATG Events	ATG Total	no ATG Events	no ATG Total	Weight	Risk Difference M-H, Random, 95% CI
**1.10.1 LOW DOSE (< 6mg/kg)**						
Chang YJ2020	10	132	4	131	17.8%	0.05 [−0.01, 0.10]
Cho BS2021	3	60	13	60	9.7%	−0.17 [−0.28, −0.05]
Walker I 2016	20	101	2	102	13.7%	0.18 [0.10, 0.26]
Subtotal (95% CI)		293		293	41.2%	0.03 [−0.13, 0.18]
Total events	33		19			
Heterogeneity: Tau² = 0.02; Chi² = 22.40, df = 2 (P < 0.0001); I² = 91%						
Test for overall effect: Z = 0.31 (P = 0.76)						
**1.10.2 HIGH DOSE (≥6 mg/kg)**						
Finke J 2009	5	103	1	99	18.9%	0.04 [−0.01, 0.08]
Kröger N 2016	3	83	1	72	18.6%	0.02 [−0.03, 0.07]
Soiffer RJ2017	2	128	0	132	21.4%	0.02 [−0.01, 0.04]
Subtotal (95% CI)		314		303	58.8%	0.02 [0.00, 0.04]
Total events	10		2			
Heterogeneity: Tau² = 0.00; Chi² = 0.83, df = 2 (P = 0.66); I² = 0%						
Test for overall effect: Z = 2.04 (P = 0.04)						
**Total (95% CI)**		607		596	100.0%	0.03 [−0.02, 0.08]
Total events	43		21			
Heterogeneity: Tau² = 0.00; Chi² = 25.68, df = 5 (P = 0.0001); I² = 81%						
Test for overall effect: Z = 1.26 (P = 0.21)						
Test for subgroup differences: Chi² = 0.00, df = 1 (P = 0.96), I² = 0%						

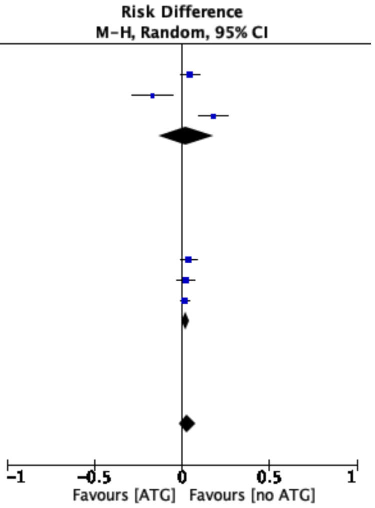

Figure 17: Analysis of the risk of EBVR and subgroup by dose - ATG (low or high dose) vs no ATG

CLINICAL QUESTION 5

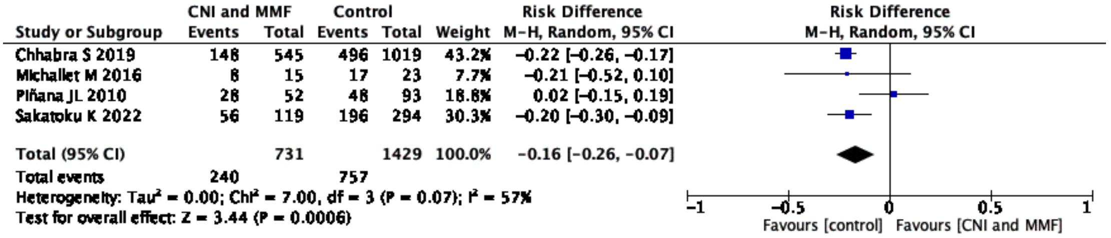

Study or Subgroup	CNI and MMF Events	CNI and MMF Total	Control Events	Control Total	Weight	Risk Difference M-H, Random, 95% CI
Chhabra S 2019	148	545	496	1019	43.2%	−0.22 [−0.26, −0.17]
Michallet M 2016	8	15	17	23	7.7%	−0.21 [−0.52, 0.10]
Piñana JL 2010	28	52	48	93	18.8%	0.02 [−0.15, 0.19]
Sakatoku K 2022	56	119	196	294	30.3%	−0.20 [−0.30, −0.09]
Total (95% CI)		731		1429	100.0%	−0.16 [−0.26, −0.07]
Total events	240		757			

Heterogeneity: Tau² = 0.00; Chi² = 7.00, df = 3 (P = 0.07); I² = 57%
Test for overall effect: Z = 3.44 (P = 0.0006)

Figure 18: Analysis of the aGVHD-free: comparison of a CNI Plus MMF vs. CNI alone or CNI plus MTX

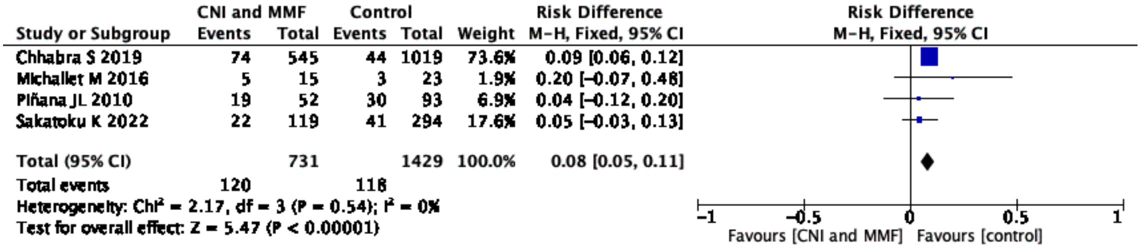

Study or Subgroup	CNI and MMF Events	CNI and MMF Total	Control Events	Control Total	Weight	Risk Difference M-H, Fixed, 95% CI
Chhabra S 2019	74	545	44	1019	73.6%	0.09 [0.06, 0.12]
Michallet M 2016	5	15	3	23	1.9%	0.20 [−0.07, 0.48]
Piñana JL 2010	19	52	30	93	6.9%	0.04 [−0.12, 0.20]
Sakatoku K 2022	22	119	41	294	17.6%	0.05 [−0.03, 0.13]
Total (95% CI)		731		1429	100.0%	0.08 [0.05, 0.11]
Total events	120		118			

Heterogeneity: Chi² = 2.17, df = 3 (P = 0.54); I² = 0%
Test for overall effect: Z = 5.47 (P < 0.00001)

Figure 19: Analysis of the aGVHD: comparison of a CNI Plus MMF vs. CNI alone or CNI plus MTX

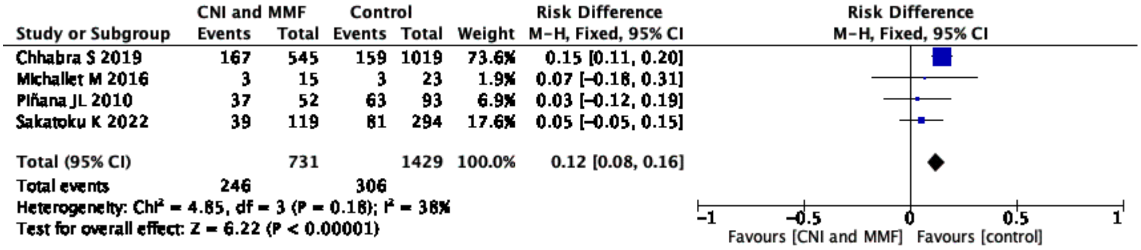

Study or Subgroup	CNI and MMF Events	CNI and MMF Total	Control Events	Control Total	Weight	Risk Difference M-H, Fixed, 95% CI
Chhabra S 2019	167	545	159	1019	73.6%	0.15 [0.11, 0.20]
Michallet M 2016	3	15	3	23	1.9%	0.07 [−0.18, 0.31]
Piñana JL 2010	37	52	63	93	6.9%	0.03 [−0.12, 0.19]
Sakatoku K 2022	39	119	81	294	17.6%	0.05 [−0.05, 0.15]
Total (95% CI)		731		1429	100.0%	0.12 [0.08, 0.16]
Total events	246		306			

Heterogeneity: Chi² = 4.85, df = 3 (P = 0.18); I² = 38%
Test for overall effect: Z = 6.22 (P < 0.00001)

Figure 20: Analysis of the cGVHD: comparison of a CNI Plus MMF vs. CNI alone or CNI plus MTX

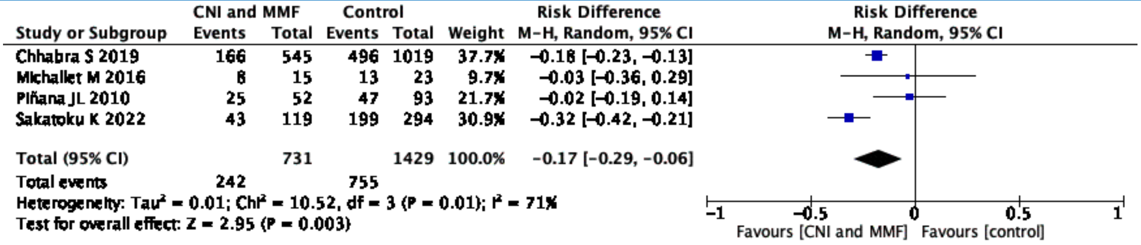

Study or Subgroup	CNI and MMF Events	CNI and MMF Total	Control Events	Control Total	Weight	Risk Difference M-H, Random, 95% CI
Chhabra S 2019	166	545	496	1019	37.7%	−0.18 [−0.23, −0.13]
Michallet M 2016	8	15	13	23	9.7%	−0.03 [−0.36, 0.29]
Piñana JL 2010	25	52	47	93	21.7%	−0.02 [−0.19, 0.14]
Sakatoku K 2022	43	119	199	294	30.9%	−0.32 [−0.42, −0.21]
Total (95% CI)		731		1429	100.0%	−0.17 [−0.29, −0.06]
Total events	242		755			

Heterogeneity: Tau² = 0.01; Chi² = 10.52, df = 3 (P = 0.01); I² = 71%
Test for overall effect: Z = 2.95 (P = 0.003)

Figure 21: Mortality analysis: comparison of a CNI Plus MMF vs. CNI alone or CNI plus MTX

Study or Subgroup	CNI and MMF Events	CNI and MMF Total	Control Events	Control Total	Weight	Risk Difference M-H, Random, 95% CI
Chhabra S 2019	127	545	145	1019	33.2%	0.09 [0.05, 0.13]
Michallet M 2016	4	15	5	23	13.6%	0.05 [−0.23, 0.33]
Piñana JL 2010	15	52	19	93	24.1%	0.08 [−0.06, 0.23]
Sakatoku K 2022	31	119	115	294	29.1%	−0.13 [−0.23, −0.03]
Total (95% CI)		731		1429	100.0%	0.02 [−0.11, 0.15]
Total events	177		284			

Heterogeneity: $Tau^2 = 0.01$; $Chi^2 = 17.32$, df = 3 (P = 0.0006); $I^2 = 83\%$
Test for overall effect: Z = 0.28 (P = 0.78)

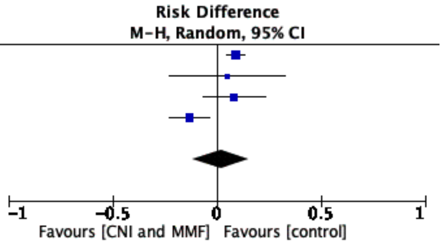

Figure 22: Analysis of the outcome “relapse or progression”: comparison of a CNI Plus MMF vs. CNI alone or CNI plus MTX

Study or Subgroup	CNI and MMF Events	CNI and MMF Total	Control Events	Control Total	Weight	Risk Difference M-H, Fixed, 95% CI
Chhabra S 2019	43	545	56	1019	75.0%	0.02 [−0.00, 0.05]
Piñana JL 2010	39	52	61	93	7.0%	0.09 [−0.06, 0.25]
Sakatoku K 2022	86	119	197	294	17.9%	0.05 [−0.04, 0.15]
Total (95% CI)		716		1406	100.0%	0.03 [0.01, 0.06]
Total events	168		314			

Heterogeneity: $Chi^2 = 1.29$, df = 2 (P = 0.52); $I^2 = 0\%$
Test for overall effect: Z = 2.34 (P = 0.02)

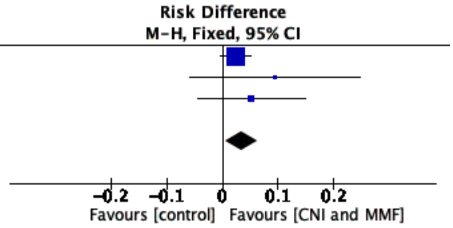

Figure 23: Analysis of the outcome “GvHD-Free and relapse-free survival” (GRFS): comparison of a CNI Plus MMF vs. CNI alone or CNI plus MTX

CLINICAL QUESTION 6

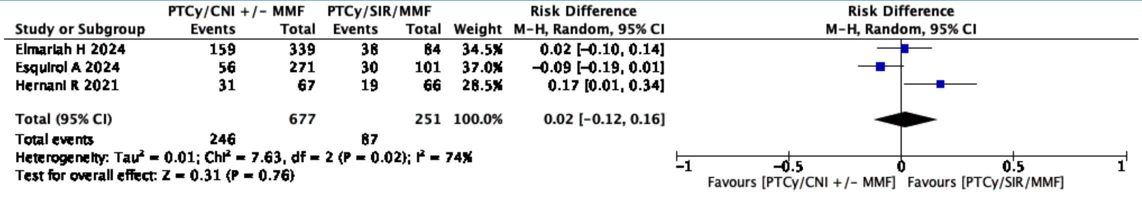

Figure 24: Analysis of the risk of aGVHD Grades II-IV: comparison of PTCy Plus CNI with or without MMF vs. PTCy Plus Sirolimus and MMF

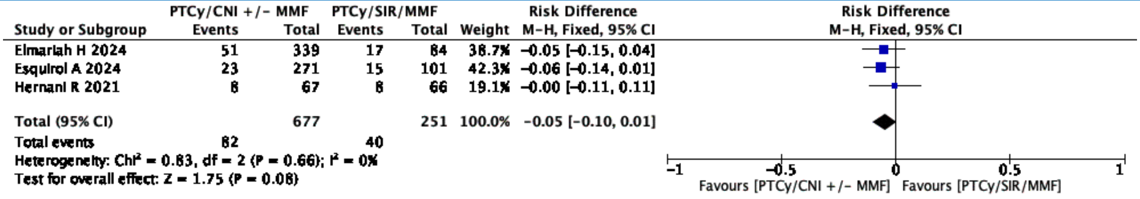

Figure 25: Analysis of the risk of aGVHD Grades III-IV: comparison of PTCy Plus CNI with or without MMF vs. PTCy Plus Sirolimus and MMF

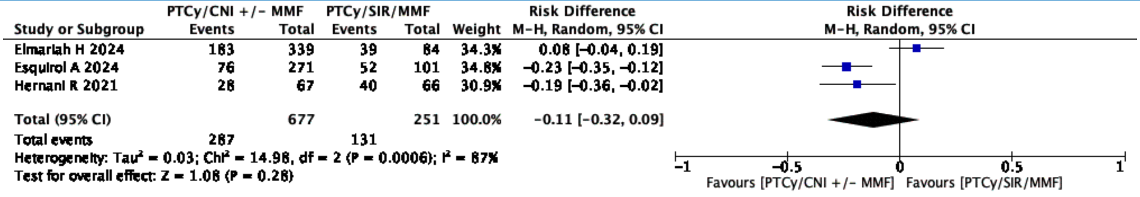

Figure 26: Analysis of the risk of cGVHD: comparison of PTCy Plus CNI with or without MMF vs. PTCy Plus Sirolimus and MMF

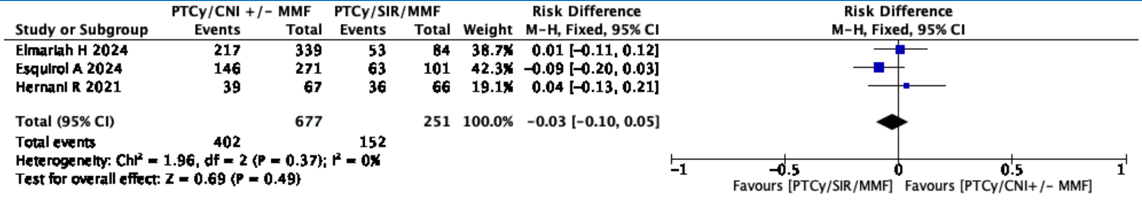

Figure 27: Analysis of OS rate: comparison of PTCy Plus CNI with or without MMF vs. PTCy Plus Sirolimus and MMF

Study or Subgroup	PTCy/CNI +/- MMF Events	PTCy/CNI +/- MMF Total	PTCy/SIR/MMF Events	PTCy/SIR/MMF Total	Weight	Risk Difference M-H, Fixed, 95% CI
Elmariah H 2024	207	339	48	84	38.7%	0.04 [-0.08, 0.16]
Esquirol A 2024	137	271	58	101	42.3%	-0.07 [-0.18, 0.04]
Hernani R 2021	34	67	40	66	19.1%	-0.10 [-0.27, 0.07]
Total (95% CI)		677		251	100.0%	-0.03 [-0.11, 0.04]
Total events	378		146			

Heterogeneity: $Chi^2 = 2.41$, df = 2 (P = 0.30); $I^2 = 17\%$
Test for overall effect: Z = 0.87 (P = 0.38)

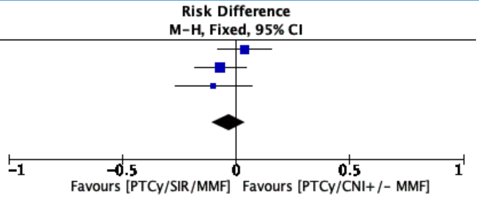

Figure 28: Analysis of EFS rate: comparison of PTCy Plus CNI with or without MMF vs. PTCy Plus Sirolimus and MMF

Study or Subgroup	PTCy/CNI +/- MMF Events	PTCy/CNI +/- MMF Total	PTCy/SIR/MMF Events	PTCy/SIR/MMF Total	Weight	Risk Difference M-H, Fixed, 95% CI
Elmariah H 2024	74	339	23	84	38.7%	-0.06 [-0.16, 0.05]
Esquirol A 2024	79	271	26	101	42.3%	0.03 [-0.07, 0.14]
Hernani R 2021	16	67	19	66	19.1%	-0.05 [-0.20, 0.10]
Total (95% CI)		677		251	100.0%	-0.02 [-0.08, 0.05]
Total events	169		68			

Heterogeneity: $Chi^2 = 1.68$, df = 2 (P = 0.43); $I^2 = 0\%$
Test for overall effect: Z = 0.49 (P = 0.62)

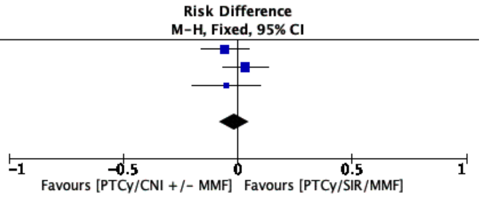

Figure 29: Analysis of the risk of NRM comparing PTCy/CI/+/-MMF with PTCy/Sirolimus/MMF

Study or Subgroup	PTCy/CNI +/- MMF Events	PTCy/CNI +/- MMF Total	PTCy/SIR/MMF Events	PTCy/SIR/MMF Total	Weight	Risk Difference M-H, Random, 95% CI
Esquirol A 2024	163	271	66	101	51.8%	-0.05 [-0.16, 0.06]
Hernani R 2021	59	67	49	66	48.2%	0.14 [0.01, 0.27]
Total (95% CI)		338		167	100.0%	0.04 [-0.15, 0.23]
Total events	222		115			

Heterogeneity: $Tau^2 = 0.02$; $Chi^2 = 4.96$, df = 1 (P = 0.03); $I^2 = 80\%$
Test for overall effect: Z = 0.41 (P = 0.68)

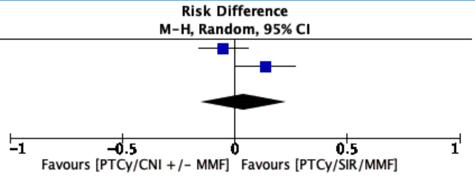

Figure 30: Analysis of CMVR: comparison of PTCy Plus CNI with or without MMF vs. PTCy Plus Sirolimus and MMF

Study or Subgroup	PTCy/CNI +/- MMF Events	PTCy/CNI +/- MMF Total	PTCy/SIR/MMF Events	PTCy/SIR/MMF Total	Weight	Risk Difference M-H, Fixed, 95% CI
Esquirol A 2024	65	271	45	101	68.9%	-0.21 [-0.32, -0.10]
Hernani R 2021	33	67	48	66	31.1%	-0.23 [-0.40, -0.07]
Total (95% CI)		338		167	100.0%	-0.21 [-0.31, -0.12]
Total events	98		93			

Heterogeneity: $Chi^2 = 0.09$, df = 1 (P = 0.77); $I^2 = 0\%$
Test for overall effect: Z = 4.65 (P < 0.00001)

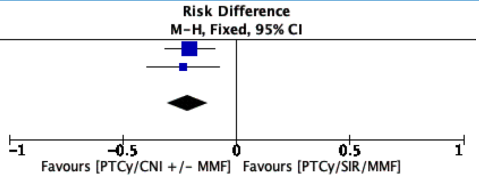

Figure 31: Analysis of the risk of HC: comparison of PTCy Plus CNI with or without MMF vs. PTCy Plus Sirolimus and MMF

Study or Subgroup	PTCy/CNI +/- MMF Events	PTCy/CNI +/- MMF Total	PTCy/SIR/MMF Events	PTCy/SIR/MMF Total	Weight	Risk Difference M-H, Fixed, 95% CI
Esquirol A 2024	11	271	13	101	68.9%	-0.09 [-0.16, -0.02]
Hernani R 2021	4	67	14	66	31.1%	-0.15 [-0.27, -0.04]
Total (95% CI)		338		167	100.0%	-0.11 [-0.17, -0.05]
Total events	15		27			

Heterogeneity: $Chi^2 = 0.90$, df = 1 (P = 0.34); $I^2 = 0\%$
Test for overall effect: Z = 3.56 (P = 0.0004)

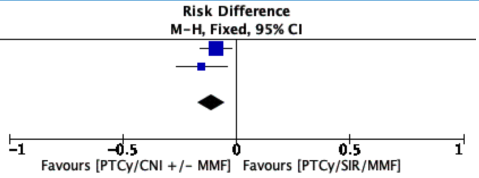

Figure 32: Analysis of the risk of SOS: comparison of PTCy Plus CNI with or without MMF vs. PTCy Plus Sirolimus and MMF

CLINICAL QUESTION 7a

Study or Subgroup	PTCy Events	PTCy Total	Without PTCy Events	Without PTCy Total	Weight	Risk Difference M-H, Random, 95% CI
Brissot E 2024	11	44	16	45	29.5%	−0.11 [−0.30, 0.08]
Broers AEC 2022	30	99	25	52	32.7%	−0.18 [−0.34, −0.01]
Luznik L 2022	43	114	35	118	37.9%	0.08 [−0.04, 0.20]
Total (95% CI)		257		215	100.0%	−0.06 [−0.23, 0.11]
Total events	84		76			

Heterogeneity: Tau² = 0.02; Chi² = 6.95, df = 2 (P = 0.03); I² = 71%
Test for overall effect: Z = 0.68 (P = 0.49)

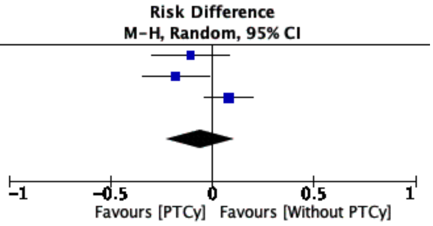

Figure 33: Analysis of the risk of aGVHD Grades II-IV: comparing PTCy-based regimens vs. non-PTCy regimens in randomized controlled trials

Study or Subgroup	PTCy Events	PTCy Total	Without PTCy Events	Without PTCy Total	Weight	Risk Difference M-H, Random, 95% CI
Brissot E 2024	2	44	3	45	24.6%	−0.02 [−0.12, 0.07]
Broers AEC 2022	6	99	6	52	24.1%	−0.05 [−0.15, 0.04]
Curtis DJ 2025	4	66	13	68	22.5%	−0.13 [−0.24, −0.02]
Luznik L 2022	12	114	4	118	28.8%	0.07 [0.01, 0.14]
Total (95% CI)		323		283	100.0%	−0.03 [−0.12, 0.06]
Total events	24		26			

Heterogeneity: Tau² = 0.01; Chi² = 11.78, df = 3 (P = 0.008); I² = 75%
Test for overall effect: Z = 0.60 (P = 0.55)

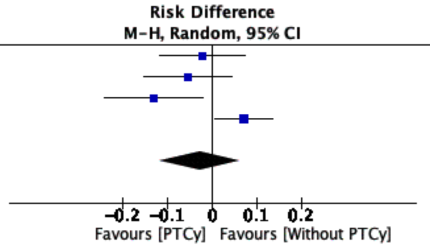

Figure 34: Analysis of the risk of aGVHD Grades III-IV: comparing PTCy-based regimens vs. non-PTCy regimens in randomized controlled trials

Study or Subgroup	PTCy Events	PTCy Total	Without PTCy Events	Without PTCy Total	Weight	Risk Difference M-H, Fixed, 95% CI
Luznik L 2022	87	114	90	118	39.2%	0.00 [−0.11, 0.11]
Curtis DJ 2025	55	66	50	68	22.7%	0.10 [−0.04, 0.24]
Broers AEC 2022	64	99	37	52	23.1%	−0.07 [−0.22, 0.09]
Brissot E 2024	35	44	36	45	15.1%	−0.00 [−0.17, 0.16]
Total (95% CI)		323		283	100.0%	0.01 [−0.06, 0.08]
Total events	241		213			

Heterogeneity: Chi² = 2.53, df = 3 (P = 0.47); I² = 0%
Test for overall effect: Z = 0.19 (P = 0.85)

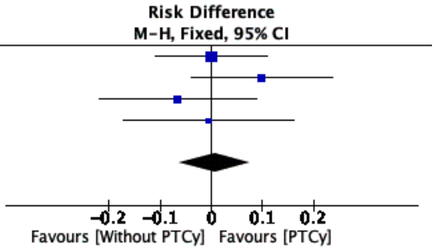

Figure 35: Analysis of OS rate: comparing PTCy-based regimens vs. non-PTCy regimens in randomized controlled trials

Study or Subgroup	PTCy Events	PTCy Total	Without PTCy Events	Without PTCy Total	Weight	Risk Difference M-H, Fixed, 95% CI
Brissot E 2024	29	44	29	45	24.8%	0.01 [−0.18, 0.21]
Broers AEC 2022	58	99	33	52	38.0%	−0.05 [−0.21, 0.11]
Curtis DJ 2025	52	66	44	68	37.3%	0.14 [−0.01, 0.29]
Total (95% CI)		209		165	100.0%	0.04 [−0.06, 0.13]
Total events	139		106			

Heterogeneity: Chi² = 2.94, df = 2 (P = 0.23); I² = 32%
Test for overall effect: Z = 0.76 (P = 0.45)

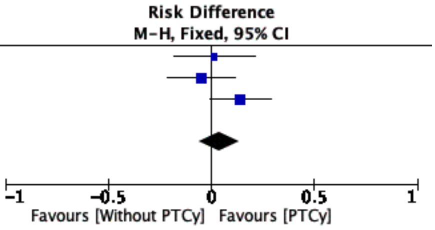

Figure 36: Analysis of EFS rate: comparing PTCy-based regimens vs. non-PTCy regimens in randomized controlled trials

Study or Subgroup	PTCy Events	PTCy Total	Without PTCy Events	Without PTCy Total	Weight	Risk Difference M-H, Random, 95% CI
Brissot E 2024	5	44	4	45	31.5%	0.02 [−0.10, 0.15]
Broers AEC 2022	10	99	7	52	34.6%	−0.03 [−0.14, 0.08]
Curtis DJ 2025	14	66	4	68	33.9%	0.15 [0.04, 0.27]
Total (95% CI)		209		165	100.0%	0.05 [−0.06, 0.16]
Total events	29		15			

Heterogeneity: $Tau^2 = 0.01$; $Chi^2 = 5.56$, df = 2 (P = 0.06); $I^2 = 64\%$
Test for overall effect: Z = 0.84 (P = 0.40)

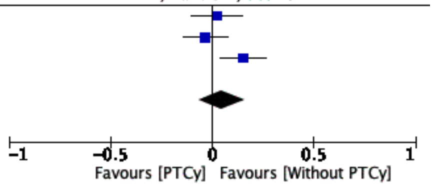

Figure 37: Analysis of the risk of NRM: comparing PTCy-based regimens vs. non-PTCy regimens in randomized controlled trials

Study or Subgroup	PTCy Events	PTCy Total	Without PTCy Events	Without PTCy Total	Weight	Risk Difference M-H, Fixed, 95% CI
Brissot E 2024	13	44	12	45	15.1%	0.03 [−0.16, 0.22]
Broers AEC 2022	1	99	0	52	23.1%	0.01 [−0.03, 0.05]
Curtis DJ 2025	14	66	10	68	22.7%	0.07 [−0.06, 0.19]
Luznik L 2022	11	114	11	118	39.2%	0.00 [−0.07, 0.08]
Total (95% CI)		323		283	100.0%	0.02 [−0.03, 0.07]
Total events	39		33			

Heterogeneity: $Chi^2 = 1.16$, df = 3 (P = 0.76); $I^2 = 0\%$
Test for overall effect: Z = 0.87 (P = 0.38)

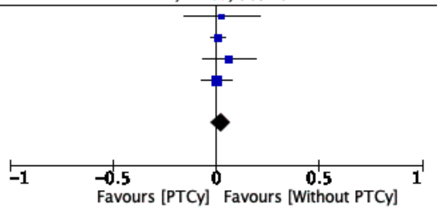

Figure 38: Analysis of CMVR: comparing PTCy-based regimens vs. non-PTCy regimens in randomized controlled trials

Study or Subgroup	PTCy Events	PTCy Total	Without PTCy Events	Without PTCy Total	Weight	Risk Difference M-H, Random, 95% CI
Brissot E 2024	5	44	8	45	31.5%	−0.06 [−0.21, 0.08]
Luznik L 2022	1	114	1	118	68.5%	0.00 [−0.02, 0.02]
Total (95% CI)		158		163	100.0%	−0.02 [−0.13, 0.09]
Total events	6		9			

Heterogeneity: $Tau^2 = 0.00$; $Chi^2 = 2.56$, df = 1 (P = 0.11); $I^2 = 61\%$
Test for overall effect: Z = 0.36 (P = 0.72)

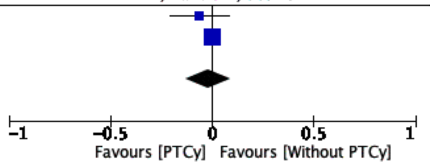

Figure 39: Analysis of EBVR: comparing PTCy-based regimens vs. non-PTCy regimens in randomized controlled trials

Study or Subgroup	PTCy Events	PTCy Total	Without PTCy Events	Without PTCy Total	Weight	Risk Difference M-H, Fixed, 95% CI
Luznik L 2022	16	44	15	45	15.1%	0.03 [−0.17, 0.23]
Curtis DJ 2025	49	99	36	52	23.1%	−0.20 [−0.36, −0.04]
Broers AEC 2022	11	66	23	68	22.7%	−0.17 [−0.32, −0.03]
Brissot E 2024	31	114	40	118	39.2%	−0.07 [−0.19, 0.05]
Total (95% CI)		323		283	100.0%	-0.11 [−0.18, −0.03]
Total events	107		114			

Heterogeneity: $Chi^2 = 4.30$, df = 3 (P = 0.23); $I^2 = 30\%$
Test for overall effect: Z = 2.82 (P = 0.005)

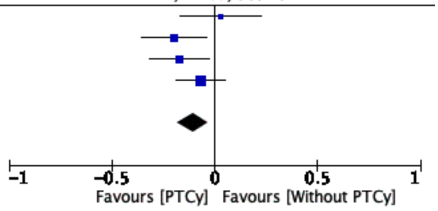

Figure 40: Analysis of the risk of cGVHD: comparing PTCy-based regimens vs. non-PTCy regimens in randomized controlled trials

Study or Subgroup	PTCy Events	PTCy Total	Without PTCy Events	Without PTCy Total	Weight	Risk Difference M-H, Random, 95% CI
Barrios JM 2020	2	10	5	10	4.8%	−0.30 [−0.70, 0.10]
Desai N 2025	29	91	85	210	21.9%	−0.09 [−0.20, 0.03]
Jacoby E 2016	0	11	5	18	10.9%	−0.28 [−0.51, −0.04]
Kwon M 2019	13	57	26	50	15.3%	−0.29 [−0.47, −0.12]
Marco-Ayala J 2023	23	100	57	100	20.6%	−0.34 [−0.47, −0.21]
Sharaf El-Deen MO 2024	4	92	24	124	26.6%	−0.15 [−0.23, −0.07]
Total (95% CI)		361		512	100.0%	-0.22 [-0.31, -0.12]
Total events	71		202			

Heterogeneity: $Tau^2 = 0.01$; $Chi^2 = 11.55$, df = 5 (P = 0.04); $I^2 = 57\%$
Test for overall effect: Z = 4.55 (P < 0.00001)

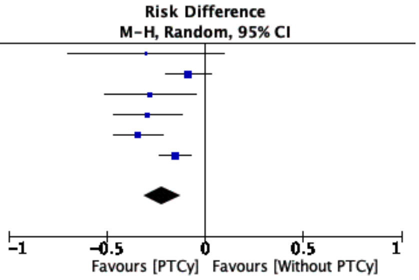

Figure 41: Analysis of the risk of aGVHD Grades II-IV: comparing PTCy-based regimens vs. non-PTCy regimens in observational cohorts

Study or Subgroup	PTCy Events	PTCy Total	Without PTCy Events	Without PTCy Total	Weight	Risk Difference M-H, Random, 95% CI
Desai N 2025	5	91	37	210	23.1%	−0.12 [−0.19, −0.05]
Jacoby E 2016	0	11	10	18	12.5%	−0.56 [−0.81, −0.31]
Kwon M 2019	5	57	12	50	19.1%	−0.15 [−0.29, −0.01]
Marco-Ayala J 2023	11	100	32	100	20.9%	−0.21 [−0.32, −0.10]
Sharaf El-Deen MO 2024	0	92	5	124	24.3%	−0.04 [−0.08, −0.00]
Total (95% CI)		351		502	100.0%	-0.18 [-0.31, -0.05]
Total events	21		96			

Heterogeneity: $Tau^2 = 0.02$; $Chi^2 = 38.11$, df = 4 (P < 0.00001); $I^2 = 90\%$
Test for overall effect: Z = 2.81 (P = 0.005)

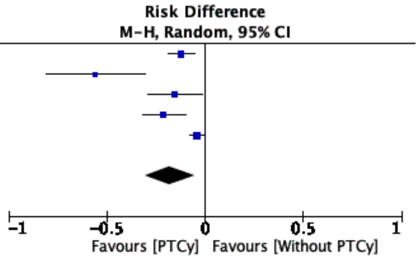

Figure 42: Analysis of the risk of aGVHD Grades III-IV: comparing PTCy-based regimens vs. non-PTCy regimens in observational cohorts

Study or Subgroup	PTCy Events	PTCy Total	Without PTCy Events	Without PTCy Total	Weight	Risk Difference M-H, Fixed, 95% CI
Barrios JM 2020	6	10	8	10	2.4%	−0.20 [−0.59, 0.19]
Desai N 2025	30	91	109	210	31.0%	−0.19 [−0.31, −0.07]
Jacoby E 2016	3	11	6	18	3.3%	−0.06 [−0.40, 0.28]
Kwon M 2019	19	57	26	50	13.0%	−0.19 [−0.37, −0.00]
Marco-Ayala J 2023	28	100	49	100	24.4%	−0.21 [−0.34, −0.08]
Sharaf El-Deen MO 2024	10	92	33	124	25.8%	−0.16 [−0.26, −0.06]
Total (95% CI)		361		512	100.0%	-0.18 [-0.24, -0.12]
Total events	96		231			

Heterogeneity: $Chi^2 = 0.91$, df = 5 (P = 0.97); $I^2 = 0\%$
Test for overall effect: Z = 5.75 (P < 0.00001)

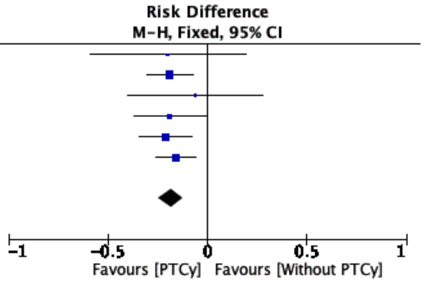

Figure 43: Analysis of the risk of cGVHD: comparing PTCy-based regimens vs. non-PTCy regimens in observational cohorts

Study or Subgroup	PTCy Events	PTCy Total	Without PTCy Events	Without PTCy Total	Weight	Risk Difference M-H, Fixed, 95% CI
Barrios JM 2020	10	10	10	10	2.4%	0.00 [−0.17, 0.17]
Desai N 2025	66	91	119	210	31.0%	0.16 [0.05, 0.27]
Jacoby E 2016	6	11	10	18	3.3%	−0.01 [−0.38, 0.36]
Kwon M 2019	44	57	28	50	13.0%	0.21 [0.04, 0.39]
Marco-Ayala J 2023	74	100	56	100	24.4%	0.18 [0.05, 0.31]
Sharaf El-Deen MO 2024	73	92	96	124	25.8%	0.02 [−0.09, 0.13]
Total (95% CI)		361		512	100.0%	0.13 [0.06, 0.19]
Total events	273		319			

Heterogeneity: $Chi^2 = 7.97$, df = 5 (P = 0.16); $I^2 = 37\%$
Test for overall effect: Z = 4.01 (P < 0.0001)

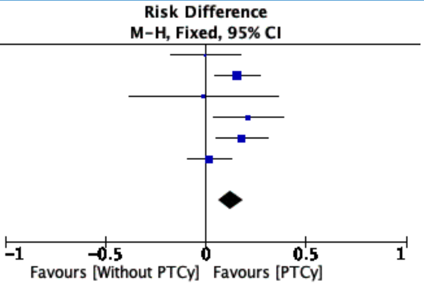

Figure 44: Analysis of OS rate: comparing PTCy-based regimens vs. non-PTCy regimens in observational cohorts

Study or Subgroup	PTCy Events	PTCy Total	Without PTCy Events	Without PTCy Total	Weight	Risk Difference M-H, Fixed, 95% CI
Barrios JM 2020	10	10	9	10	3.5%	0.10 [-0.14, 0.34]
Jacoby E 2016	5	11	8	18	4.8%	0.01 [-0.36, 0.38]
Kwon M 2019	36	57	24	50	18.9%	0.15 [-0.04, 0.34]
Marco-Ayala J 2023	65	100	47	100	35.4%	0.18 [0.04, 0.32]
Sharaf El-Deen MO 2024	62	92	89	124	37.4%	-0.04 [-0.17, 0.08]
Total (95% CI)		270		302	100.0%	0.08 [0.00, 0.16]
Total events	178		177			

Heterogeneity: $Chi^2 = 6.64$, df = 4 ($P = 0.16$); $I^2 = 40\%$
Test for overall effect: $Z = 2.01$ ($P = 0.04$)

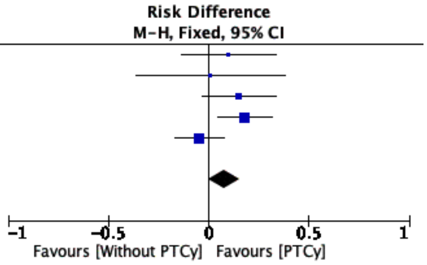

Figure 45: Analysis of EFS rate: comparing PTCy-based regimens vs. non-PTCy regimens in observational cohorts

Study or Subgroup	PTCy Events	PTCy Total	Without PTCy Events	Without PTCy Total	Weight	Risk Difference M-H, Fixed, 95% CI
Desai N 2025	2	91	9	210	31.8%	-0.02 [-0.06, 0.02]
Jacoby E 2016	0	11	2	18	3.4%	-0.11 [-0.30, 0.08]
Kwon M 2019	5	57	12	50	13.3%	-0.15 [-0.29, -0.01]
Marco-Ayala J 2023	14	100	21	100	25.0%	-0.07 [-0.17, 0.03]
Sharaf El-Deen MO 2024	15	92	21	124	26.4%	-0.01 [-0.11, 0.09]
Total (95% CI)		351		502	100.0%	-0.05 [-0.09, -0.01]
Total events	36		65			

Heterogeneity: $Chi^2 = 5.30$, df = 4 ($P = 0.26$); $I^2 = 24\%$
Test for overall effect: $Z = 2.21$ ($P = 0.03$)

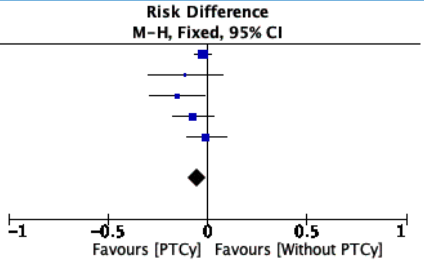

Figure 46: Analysis of the risk of NRM: comparing PTCy-based regimens vs. non-PTCy regimens in observational cohorts

Study or Subgroup	PTCy Events	PTCy Total	Without PTCy Events	Without PTCy Total	Weight	Risk Difference M-H, Random, 95% CI
Desai N 2025	45	91	32	210	27.3%	0.34 [0.23, 0.46]
Kwon M 2019	27	57	12	50	20.5%	0.23 [0.06, 0.41]
Marco-Ayala J 2023	39	100	11	100	27.3%	0.28 [0.17, 0.39]
Sharaf El-Deen MO 2024	49	92	58	124	24.9%	0.06 [-0.07, 0.20]
Total (95% CI)		340		484	100.0%	0.23 [0.11, 0.35]
Total events	160		113			

Heterogeneity: $Tau^2 = 0.01$; $Chi^2 = 10.15$, df = 3 ($P = 0.02$); $I^2 = 70\%$
Test for overall effect: $Z = 3.79$ ($P = 0.0001$)

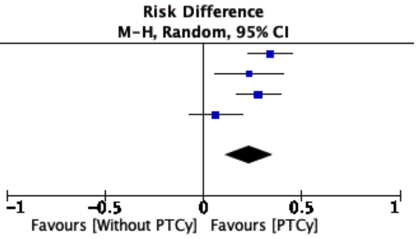

Figure 47: Analysis of the risk of GRFS: comparing PTCy-based regimens vs. non-PTCy regimens in observational cohorts

Study or Subgroup	PTCy Events	PTCy Total	Without PTCy Events	Without PTCy Total	Weight	Risk Difference M-H, Fixed, 95% CI
Jacoby E 2016	3	11	2	18	8.2%	0.16 [-0.14, 0.46]
Kwon M 2019	8	57	4	50	31.9%	0.06 [-0.06, 0.18]
Marco-Ayala J 2023	34	100	12	100	59.9%	0.22 [0.11, 0.33]
Total (95% CI)		168		168	100.0%	0.16 [0.08, 0.25]
Total events	45		18			

Heterogeneity: $Chi^2 = 3.95$, df = 2 ($P = 0.14$); $I^2 = 49\%$
Test for overall effect: $Z = 3.98$ ($P < 0.0001$)

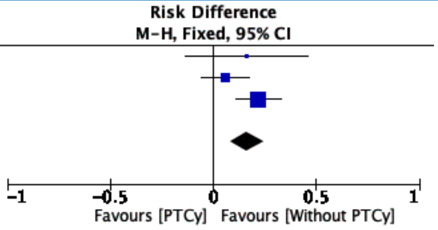

Figure 48: Analysis of the risk of HC: comparing PTCy-based regimens vs. non-PTCy regimens in observational cohorts

Study or Subgroup	PTCy Events	PTCy Total	Without PTCy Events	Without PTCy Total	Weight	Risk Difference M-H, Random, 95% CI
Kwon M 2019	0	57	2	50	49.4%	−0.04 [−0.10, 0.02]
Marco-Ayala J 2023	8	100	2	100	50.6%	0.06 [0.00, 0.12]
Total (95% CI)		157		150	100.0%	0.01 [−0.09, 0.11]
Total events	8		4			

Heterogeneity: $Tau^2 = 0.00$; $Chi^2 = 5.31$, df = 1 (P = 0.02); $I^2 = 81\%$
Test for overall effect: Z = 0.21 (P = 0.84)

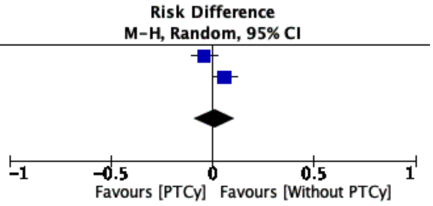

Figure 49: Analysis of the risk of SOS: comparing PTCy-based regimens vs. non-PTCy regimens in observational cohorts

Study or Subgroup	PTCy Events	PTCy Total	Without PTCy Events	Without PTCy Total	Weight	Risk Difference M-H, Fixed, 95% CI
Desai N 2025	20	91	61	210	65.5%	−0.07 [−0.18, 0.03]
Jacoby E 2016	0	11	3	18	7.0%	−0.17 [−0.37, 0.04]
Kwon M 2019	28	57	20	50	27.5%	0.09 [−0.10, 0.28]
Total (95% CI)		159		278	100.0%	−0.03 [−0.12, 0.05]
Total events	48		84			

Heterogeneity: $Chi^2 = 3.77$, df = 2 (P = 0.15); $I^2 = 47\%$
Test for overall effect: Z = 0.74 (P = 0.46)

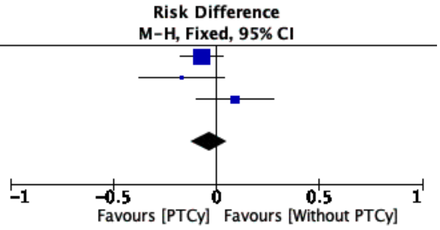

Figure 50: Analysis of CMVR: comparing PTCy-based regimens vs. non-PTCy regimens in observational cohorts

Study or Subgroup	PTCy + CNI Events	PTCy + CNI Total	PTCy + CNI + MMF Events	PTCy + CNI + MMF Total	Weight	Risk Difference M-H, Random, 95% CI
Battipaglia G 2025	66	234	50	250	50.4%	0.08 [0.01, 0.16]
Mehta RS 2022	92	242	96	144	49.6%	−0.29 [−0.38, −0.19]
Total (95% CI)		476		394	100.0%	−0.10 [−0.46, 0.26]
Total events	158		146			

Heterogeneity: $Tau^2 = 0.07$; $Chi^2 = 34.18$, df = 1 (P < 0.00001); $I^2 = 97\%$
Test for overall effect: Z = 0.54 (P = 0.59)

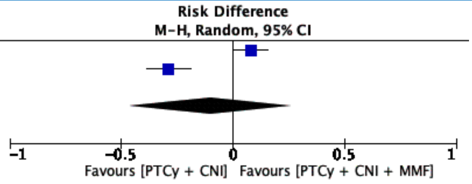

Figure 51: Analysis of the risk of aGVHD Grades II-IV: comparing PTCy-based regimes with and without MMF in observational cohorts

Study or Subgroup	PTCy + CNI Events	PTCy + CNI Total	PTCy + CNI + MMF Events	PTCy + CNI + MMF Total	Weight	Risk Difference M-H, Fixed, 95% CI
Battipaglia G 2025	14	234	22	250	57.2%	−0.03 [−0.07, 0.02]
Mehta RS 2022	19	242	14	144	42.8%	−0.02 [−0.08, 0.04]
Total (95% CI)		476		394	100.0%	−0.02 [−0.06, 0.01]
Total events	33		36			

Heterogeneity: $Chi^2 = 0.06$, df = 1 (P = 0.80); $I^2 = 0\%$
Test for overall effect: Z = 1.29 (P = 0.20)

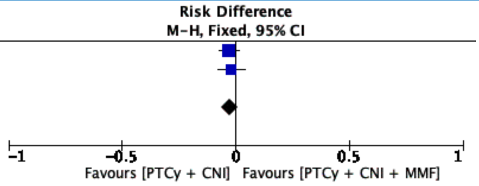

Figure 52: Analysis of the risk of aGVHD Grades III-IV: comparing PTCy-based regimes with and without MMF in observational cohorts

Study or Subgroup	PTCy + CNI Events	PTCy + CNI Total	PTCy + CNI + MMF Events	PTCy + CNI + MMF Total	Weight	Risk Difference M-H, Random, 95% CI
Battipaglia G 2025	37	234	15	250	52.1%	0.10 [0.04, 0.15]
Mehta RS 2022	34	242	24	144	47.9%	−0.03 [−0.10, 0.05]
Total (95% CI)		476		394	100.0%	0.04 [−0.08, 0.16]
Total events	71		39			

Heterogeneity: $Tau^2 = 0.01$; $Chi^2 = 7.01$, df = 1 (P = 0.008); $I^2 = 86\%$
Test for overall effect: Z = 0.61 (P = 0.54)

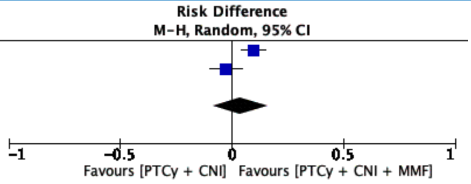

Figure 53: Analysis of the risk of cGVHD: comparing PTCy-based regimes with and without MMF in observational cohorts

Study or Subgroup	PTCy + CNI Events	PTCy + CNI Total	PTCy + CNI + MMF Events	PTCy + CNI + MMF Total	Weight	Risk Difference M-H, Fixed, 95% CI
Battipaglia G 2025	157	234	160	250	57.2%	0.03 [−0.05, 0.12]
Mehta RS 2022	165	242	99	144	42.8%	−0.01 [−0.10, 0.09]
Total (95% CI)		476		394	100.0%	0.02 [−0.05, 0.08]
Total events	322		259			

Heterogeneity: $Chi^2 = 0.32$, df = 1 (P = 0.57); $I^2 = 0\%$
Test for overall effect: Z = 0.47 (P = 0.64)

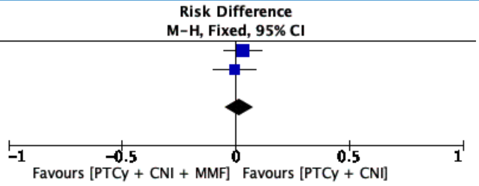

Figure 54: Analysis of OS rate: comparing PTCy-based regimes with and without MMF in observational cohorts

Study or Subgroup	PTCy + CNI Events	PTCy + CNI Total	PTCy + CNI + MMF Events	PTCy + CNI + MMF Total	Weight	Risk Difference M-H, Random, 95% CI
Battipaglia G 2025	145	234	150	250	51.5%	0.02 [−0.07, 0.11]
Mehta RS 2022	136	242	98	144	48.5%	−0.12 [−0.22, −0.02]
Total (95% CI)		476		394	100.0%	-0.05 [−0.18, 0.09]
Total events	281		248			

Heterogeneity: $Tau^2 = 0.01$; $Chi^2 = 4.25$, df = 1 (P = 0.04); $I^2 = 76\%$
Test for overall effect: Z = 0.69 (P = 0.49)

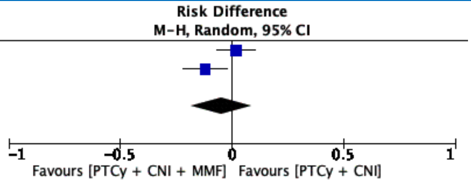

Figure 55: Analysis of EFS rate: comparing PTCy-based regimes with and without MMF in observational cohorts

Study or Subgroup	PTCy + CNI Events	PTCy + CNI Total	PTCy + CNI + MMF Events	PTCy + CNI + MMF Total	Weight	Risk Difference M-H, Fixed, 95% CI
Battipaglia G 2025	19	234	32	250	57.2%	−0.05 [−0.10, 0.01]
Mehta RS 2022	24	242	19	144	42.8%	−0.03 [−0.10, 0.03]
Total (95% CI)		476		394	100.0%	-0.04 [−0.08, 0.00]
Total events	43		51			

Heterogeneity: $Chi^2 = 0.10$, df = 1 (P = 0.75); $I^2 = 0\%$
Test for overall effect: Z = 1.89 (P = 0.06)

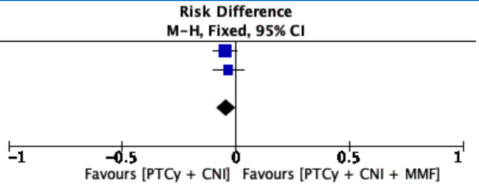

Figure 56: Analysis the risk of NRM: comparing PTCy-based regimes with and without MMF in observational cohorts

CLINICAL QUESTION 7b

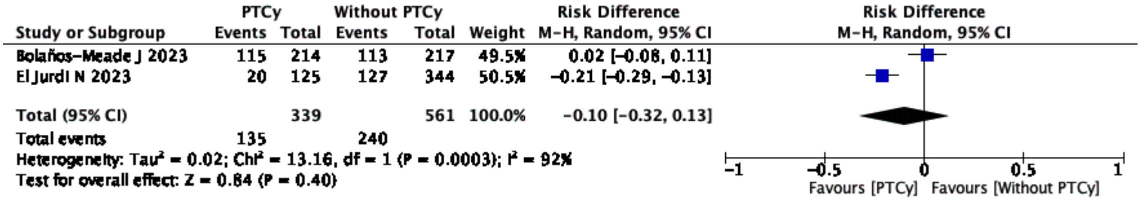

Figure 57: Analysis of the risk of aGVHD Grades II-IV: comparing PTCy-based regimens vs. non-PTCy regimens in randomized controlled trials

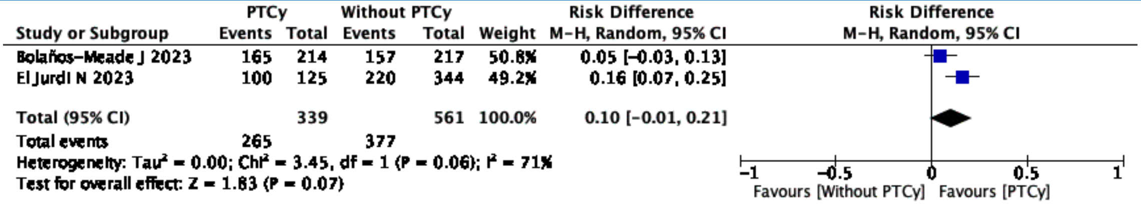

Figure 58: Analysis of Overall Survival (OS) rate: comparing PTCy-based regimens vs. non-PTCy regimens in randomized controlled trials

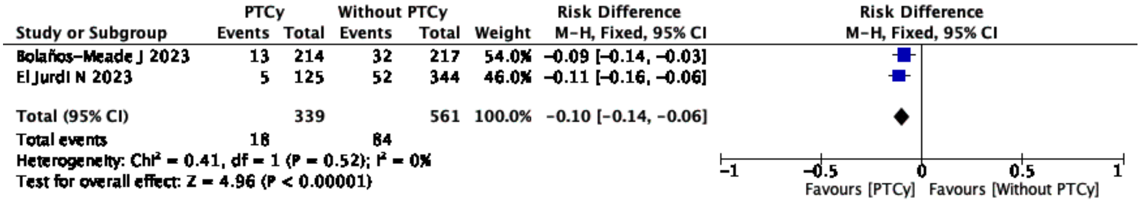

Figure 59: Analysis of the risk of aGVHD Grades III-IV: comparing PTCy-based regimens vs. non-PTCy regimens in randomized controlled trials

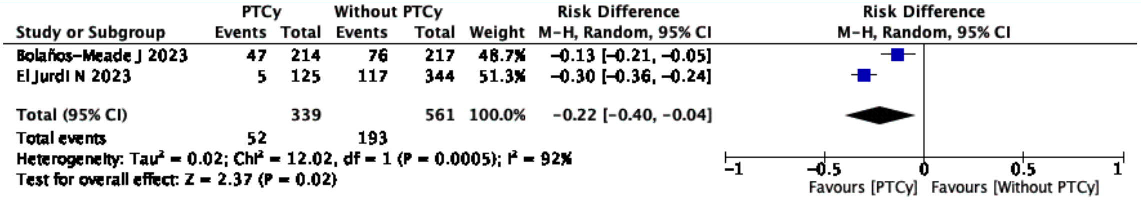

Figure 60: Analysis of the risk of cGVHD: comparing PTCy-based regimens vs. non-PTCy regimens in randomized controlled trials

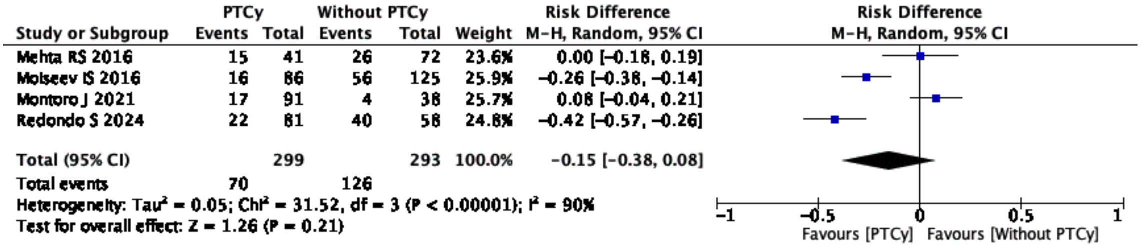

Study or Subgroup	PTCy Events	PTCy Total	Without PTCy Events	Without PTCy Total	Weight	Risk Difference M-H, Random, 95% CI
Mehta RS 2016	15	41	26	72	23.6%	0.00 [−0.18, 0.19]
Moiseev IS 2016	16	86	56	125	25.9%	−0.26 [−0.38, −0.14]
Montoro J 2021	17	91	4	38	25.7%	0.08 [−0.04, 0.21]
Redondo S 2024	22	81	40	58	24.8%	−0.42 [−0.57, −0.26]
Total (95% CI)		299		293	100.0%	−0.15 [−0.38, 0.08]
Total events	70		126			

Heterogeneity: Tau² = 0.05; Chi² = 31.52, df = 3 (P < 0.00001); I² = 90%
Test for overall effect: Z = 1.26 (P = 0.21)

Figure 61: Analysis of the risk of aGVHD Grades II-IV: comparing PTCy-based regimens vs. non-PTCy regimens in observational cohorts

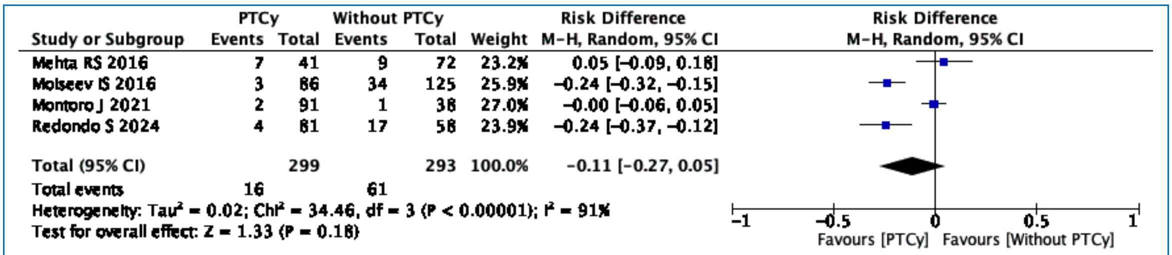

Study or Subgroup	PTCy Events	PTCy Total	Without PTCy Events	Without PTCy Total	Weight	Risk Difference M-H, Random, 95% CI
Mehta RS 2016	7	41	9	72	23.2%	0.05 [−0.09, 0.18]
Moiseev IS 2016	3	86	34	125	25.9%	−0.24 [−0.32, −0.15]
Montoro J 2021	2	91	1	38	27.0%	−0.00 [−0.06, 0.05]
Redondo S 2024	4	81	17	58	23.9%	−0.24 [−0.37, −0.12]
Total (95% CI)		299		293	100.0%	−0.11 [−0.27, 0.05]
Total events	16		61			

Heterogeneity: Tau² = 0.02; Chi² = 34.46, df = 3 (P < 0.00001); I² = 91%
Test for overall effect: Z = 1.33 (P = 0.18)

Figure 62: Analysis of the risk of aGVHD Grades III-IV: comparing PTCy-based regimens vs. non-PTCy regimens in observational cohorts

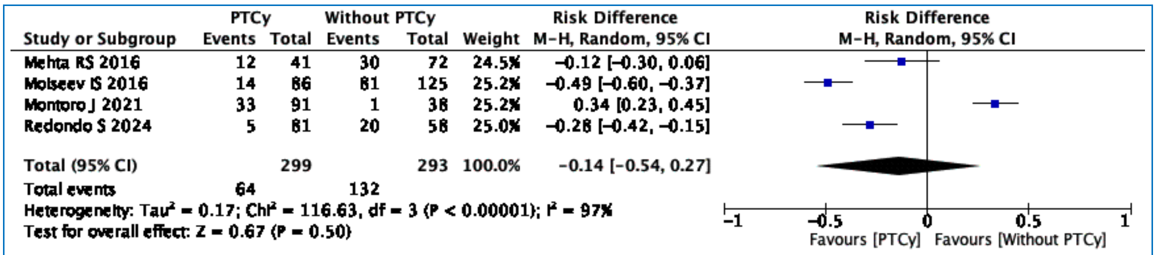

Study or Subgroup	PTCy Events	PTCy Total	Without PTCy Events	Without PTCy Total	Weight	Risk Difference M-H, Random, 95% CI
Mehta RS 2016	12	41	30	72	24.5%	−0.12 [−0.30, 0.06]
Moiseev IS 2016	14	86	81	125	25.2%	−0.49 [−0.60, −0.37]
Montoro J 2021	33	91	1	38	25.2%	0.34 [0.23, 0.45]
Redondo S 2024	5	81	20	58	25.0%	−0.28 [−0.42, −0.15]
Total (95% CI)		299		293	100.0%	−0.14 [−0.54, 0.27]
Total events	64		132			

Heterogeneity: Tau² = 0.17; Chi² = 116.63, df = 3 (P < 0.00001); I² = 97%
Test for overall effect: Z = 0.67 (P = 0.50)

Figure 63: Analysis of the risk of cGVHD: comparing PTCy-based regimens vs. non-PTCy regimens in observational cohorts

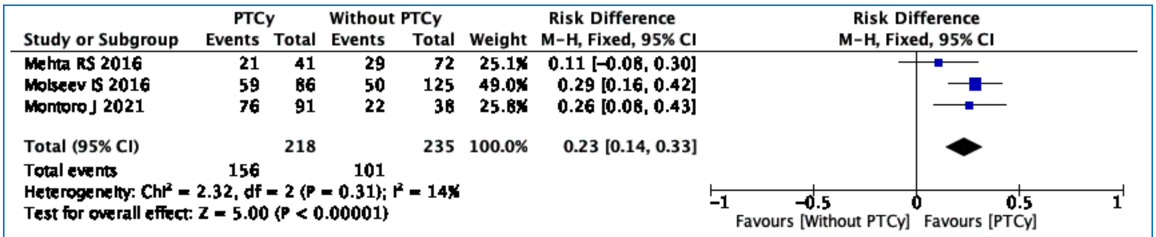

Study or Subgroup	PTCy Events	PTCy Total	Without PTCy Events	Without PTCy Total	Weight	Risk Difference M-H, Fixed, 95% CI
Mehta RS 2016	21	41	29	72	25.1%	0.11 [−0.08, 0.30]
Moiseev IS 2016	59	86	50	125	49.0%	0.29 [0.16, 0.42]
Montoro J 2021	76	91	22	38	25.8%	0.26 [0.08, 0.43]
Total (95% CI)		218		235	100.0%	0.23 [0.14, 0.33]
Total events	156		101			

Heterogeneity: Chi² = 2.32, df = 2 (P = 0.31); I² = 14%
Test for overall effect: Z = 5.00 (P < 0.00001)

Figure 64: Analysis of OS rate: comparing PTCy-based regimens vs. non-PTCy regimens in observational cohorts

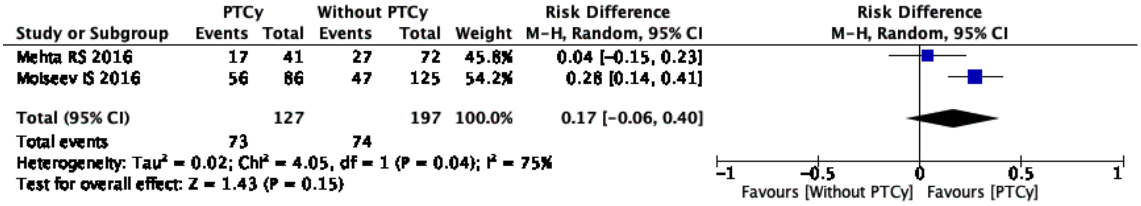

Figure 65: Analysis of EFS rate: comparing PTCy-based regimens vs. non-PTCy regimens in observational cohorts

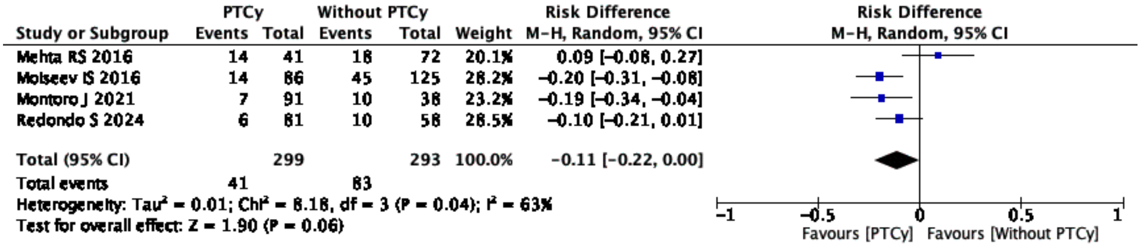

Figure 66: Analysis of the risk of NRM: comparing PTCy-based regimens vs. non-PTCy regimens in observational cohorts

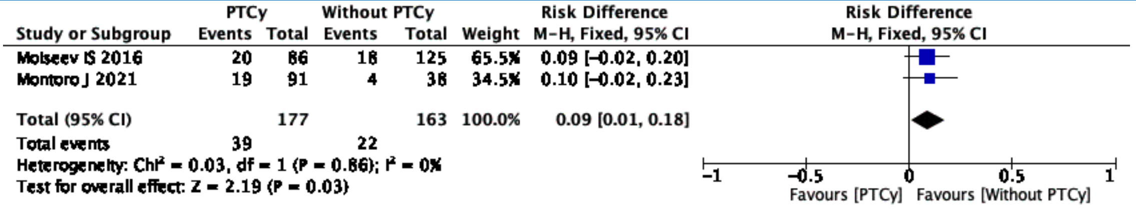

Figure 67: Analysis of the risk of HC: comparing PTCy-based regimens vs. non-PTCy regimens in observational cohorts

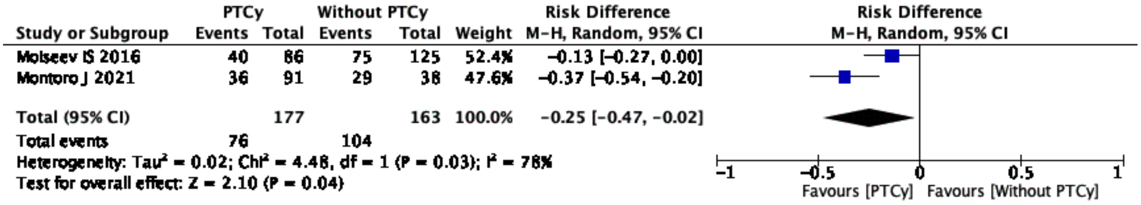

Figure 68: Analysis of the risk of CMVR: comparing PTCy-based regimens vs. non-PTCy regimens in observational cohorts

Study or Subgroup	PTCy +ATG Events	PTCy +ATG Total	Control Events	Control Total	Weight	Risk Difference M-H, Random, 95% CI
**1.1.1 HAPLOIDENTICAL**						
Bazarbachi AH 2024	57	292	825	2999	20.2%	−0.08 [−0.13, −0.03]
El-Cheikh J 2020	8	69	44	199	15.3%	−0.11 [−0.20, −0.01]
Makanga DR 2020	6	26	19	32	5.8%	−0.36 [−0.60, −0.13]
Pasic I 2020	7	62	20	71	11.9%	−0.17 [−0.30, −0.04]
Subtotal (95% CI)		449		3301	53.1%	-0.13 [-0.21, -0.05]
Total events	78		908			
Heterogeneity: Tau² = 0.00; Chi² = 6.56, df = 3 (P = 0.09); I² = 54%						
Test for overall effect: Z = 3.30 (P = 0.0010)						
**1.1.2 NOT HAPLOIDENTICAL**						
Desai N 2025	12	78	29	91	12.4%	−0.16 [−0.29, −0.04]
Kunacheewa C 2020	4	21	5	21	5.3%	−0.05 [−0.30, 0.20]
Salas MQ 2024	30	84	30	127	12.3%	0.12 [−0.01, 0.25]
Spyridonidis A 2022	35	151	122	421	16.9%	−0.06 [−0.14, 0.02]
Subtotal (95% CI)		334		660	46.9%	-0.04 [-0.16, 0.08]
Total events	81		186			
Heterogeneity: Tau² = 0.01; Chi² = 10.32, df = 3 (P = 0.02); I² = 71%						
Test for overall effect: Z = 0.61 (P = 0.54)						
**Total (95% CI)**		783		3961	100.0%	-0.09 [-0.16, -0.03]
Total events	159		1094			
Heterogeneity: Tau² = 0.00; Chi² = 19.50, df = 7 (P = 0.007); I² = 64%						
Test for overall effect: Z = 2.73 (P = 0.006)						
Test for subgroup differences: Chi² = 1.63, df = 1 (P = 0.20), I² = 38.7%						

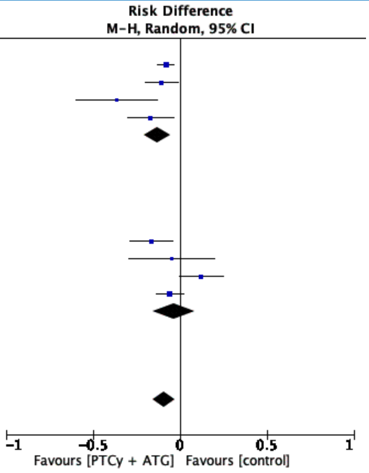

Figure 69: Analysis of aGVHD Grades II–IV: comparing PTCy plus ATG vs. other regimens

Study or Subgroup	PTCy +ATG Events	PTCy +ATG Total	Control Events	Control Total	Weight	Risk Difference M-H, Random, 95% CI
**1.2.1 HAPLOIDENTICAL**						
Bazarbachi AH 2024	20	292	288	2999	21.6%	−0.03 [−0.06, 0.00]
Chen TT 2022	8	61	1	22	8.2%	0.09 [−0.04, 0.21]
Makanga DR 2020	1	26	6	32	5.9%	−0.15 [−0.30, 0.01]
Pasic I 2020	0	62	5	71	15.6%	−0.07 [−0.14, −0.01]
Subtotal (95% CI)		441		3124	51.2%	-0.04 [-0.10, 0.02]
Total events	29		300			
Heterogeneity: Tau² = 0.00; Chi² = 7.24, df = 3 (P = 0.06); I² = 59%						
Test for overall effect: Z = 1.19 (P = 0.24)						
**1.2.2 NOT HAPLOIDENTICAL**						
Desai N 2025	2	78	5	91	16.7%	−0.03 [−0.09, 0.03]
Kunacheewa C 2020	2	21	4	21	3.6%	−0.10 [−0.30, 0.11]
Salas MQ 2024	18	84	10	127	10.5%	0.14 [0.04, 0.24]
Spyridonidis A 2022	12	151	40	421	18.0%	−0.02 [−0.07, 0.04]
Subtotal (95% CI)		334		660	48.8%	0.01 [-0.07, 0.08]
Total events	34		59			
Heterogeneity: Tau² = 0.00; Chi² = 10.10, df = 3 (P = 0.02); I² = 70%						
Test for overall effect: Z = 0.21 (P = 0.84)						
**Total (95% CI)**		775		3784	100.0%	-0.02 [-0.06, 0.03]
Total events	63		359			
Heterogeneity: Tau² = 0.00; Chi² = 18.71, df = 7 (P = 0.009); I² = 63%						
Test for overall effect: Z = 0.70 (P = 0.48)						
Test for subgroup differences: Chi² = 0.81, df = 1 (P = 0.37), I² = 0%						

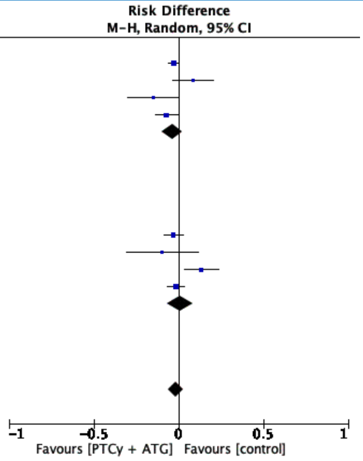

Figure 70: Analysis of aGVHD Grades III–IV: comparing PTCy plus ATG vs. other regimens

Study or Subgroup	PTCy +ATG Events	PTCy +ATG Total	Control Events	Control Total	Weight	Risk Difference M-H, Random, 95% CI
**1.3.1 HAPLOIDENTICAL**						
Bazarbachi AH 2024	87	292	942	2999	15.6%	−0.02 [−0.07, 0.04]
Chen TT 2022	10	61	4	22	8.7%	−0.02 [−0.20, 0.17]
El-Cheikh J 2020	16	69	42	199	12.4%	0.02 [−0.09, 0.14]
Makanga DR 2020	3	26	7	32	8.6%	−0.10 [−0.29, 0.09]
Pasic I 2020	16	62	33	71	10.0%	−0.21 [−0.37, −0.05]
Subtotal (95% CI)		510		3323	55.2%	−0.04 [−0.11, 0.02]
Total events	132		1028			
Heterogeneity: Tau² = 0.00; Chi² = 6.33, df = 4 (P = 0.18); I² = 37%						
Test for overall effect: Z = 1.26 (P = 0.21)						
**1.3.2 NOT HAPLOIDENTICAL**						
Desai N 2025	17	78	39	91	11.2%	−0.21 [−0.35, −0.07]
Kunacheewa C 2020	4	21	16	21	6.3%	−0.57 [−0.82, −0.32]
Salas MQ 2024	12	84	18	127	13.4%	0.00 [−0.10, 0.10]
Spyridonidis A 2022	50	151	147	421	13.9%	−0.02 [−0.11, 0.07]
Subtotal (95% CI)		334		660	44.8%	−0.17 [−0.34, 0.01]
Total events	83		220			
Heterogeneity: Tau² = 0.03; Chi² = 23.23, df = 3 (P < 0.0001); I² = 87%						
Test for overall effect: Z = 1.87 (P = 0.06)						
Total (95% CI)		844		3983	100.0%	−0.09 [−0.17, −0.01]
Total events	215		1248			
Heterogeneity: Tau² = 0.01; Chi² = 30.94, df = 8 (P = 0.0001); I² = 74%						
Test for overall effect: Z = 2.31 (P = 0.02)						
Test for subgroup differences: Chi² = 1.60, df = 1 (P = 0.21), I² = 37.5%						

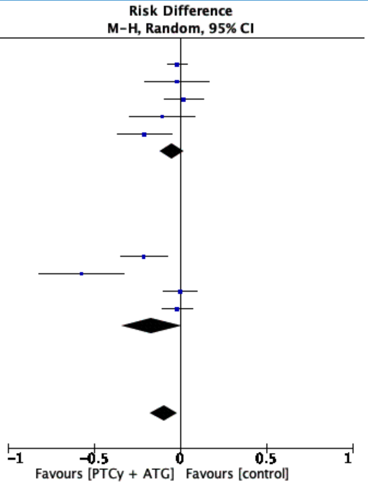

Figure 71: Analysis of cGVHD: comparing PTCy plus ATG vs. other regimens

Study or Subgroup	PTCy +ATG Events	PTCy +ATG Total	Control Events	Control Total	Weight	Risk Difference M-H, Fixed, 95% CI
**1.8.1 HAPLOIDENTICAL**						
Bazarbachi AH 2024	184	292	1874	2999	44.7%	0.01 [−0.05, 0.06]
Chen TT 2022	26	61	6	22	2.7%	0.15 [−0.07, 0.38]
El-Cheikh J 2020	55	69	137	199	8.6%	0.11 [−0.01, 0.22]
Makanga DR 2020	16	26	23	32	2.4%	−0.10 [−0.35, 0.14]
Pasic I 2020	35	62	26	71	5.6%	0.20 [0.03, 0.37]
Subtotal (95% CI)		510		3323	64.0%	0.04 [−0.01, 0.09]
Total events	316		2066			
Heterogeneity: Chi² = 8.55, df = 4 (P = 0.07); I² = 53%						
Test for overall effect: Z = 1.57 (P = 0.12)						
**1.8.2 NOT HAPLOIDENTICAL**						
Desai N 2025	48	78	66	91	7.1%	−0.11 [−0.25, 0.03]
Kunacheewa C 2020	12	21	15	21	1.8%	−0.14 [−0.43, 0.14]
Salas MQ 2024	59	84	92	127	8.5%	−0.02 [−0.15, 0.10]
Spyridonidis A 2022	101	151	294	421	18.7%	−0.03 [−0.12, 0.06]
Subtotal (95% CI)		334		660	36.0%	−0.05 [−0.11, 0.01]
Total events	220		467			
Heterogeneity: Chi² = 1.49, df = 3 (P = 0.68); I² = 0%						
Test for overall effect: Z = 1.54 (P = 0.12)						
Total (95% CI)		844		3983	100.0%	0.01 [−0.03, 0.04]
Total events	536		2533			
Heterogeneity: Chi² = 15.07, df = 8 (P = 0.06); I² = 47%						
Test for overall effect: Z = 0.35 (P = 0.73)						
Test for subgroup differences: Chi² = 4.76, df = 1 (P = 0.03), I² = 79.0%						

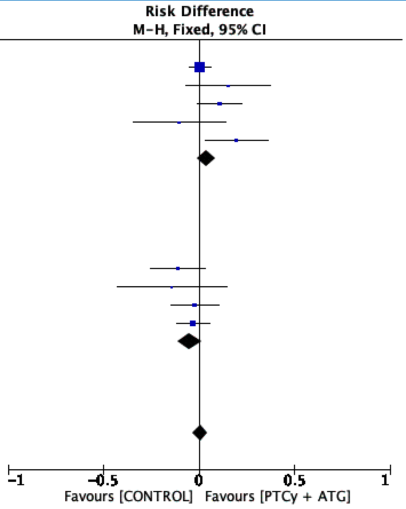

Figure 72: Analysis of OS: comparing PTCy plus ATG vs. other regimens

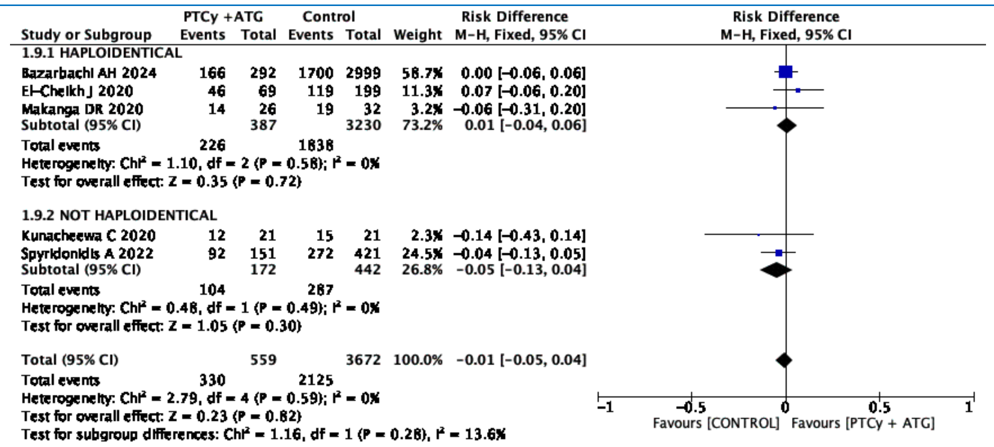

Figure 73: Analysis of EFS: comparing PTCy plus ATG vs. other regimens

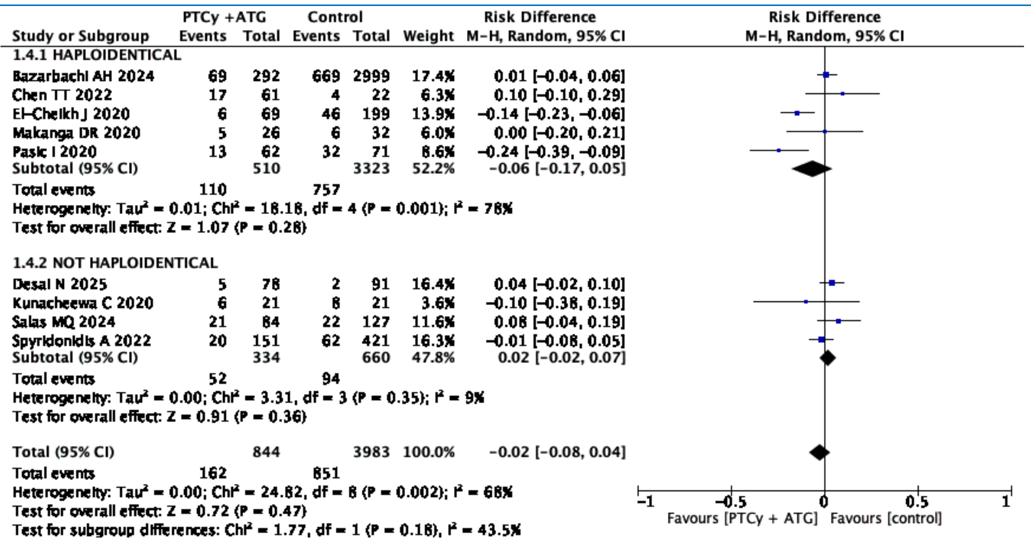

Figure 74: Analysis of NRM: comparing PTCy plus ATG vs. other regimens

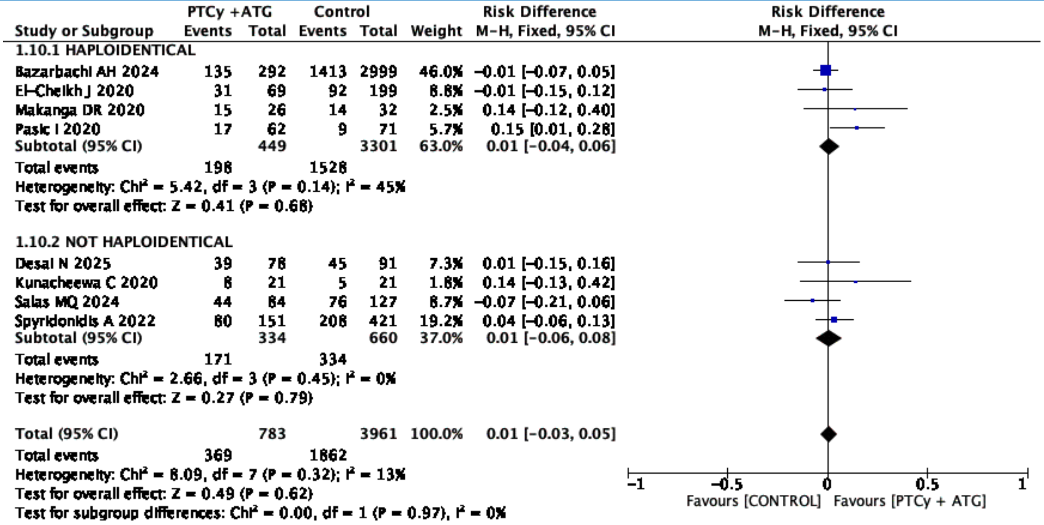

Figure 75: Analysis of GRFS: comparing PTCy plus ATG vs. other regimens

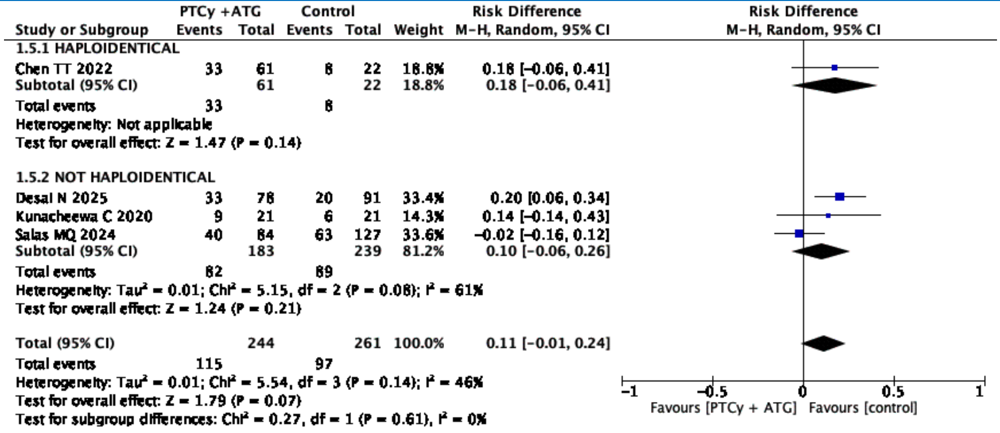

Figure 76: Analysis of CMVR: comparing PTCy plus ATG vs. other regimens

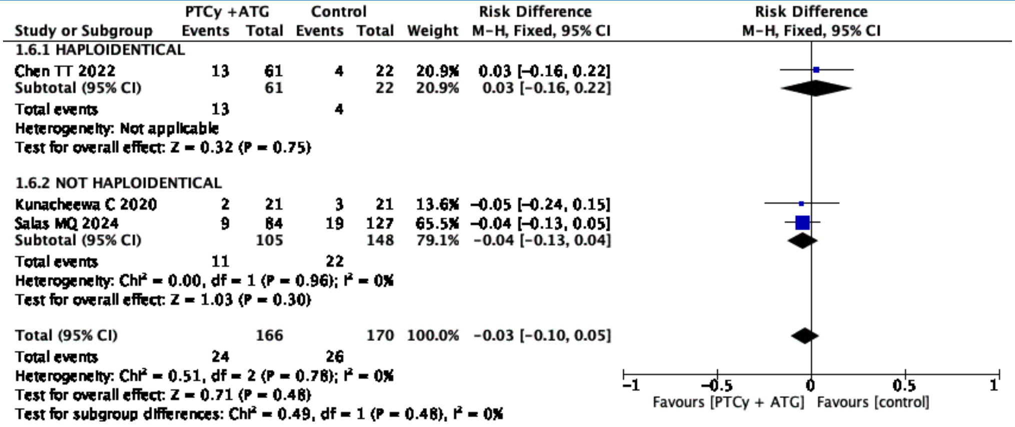

Figure 77: Analysis of HC: comparing PTCy plus ATG vs. other regimens

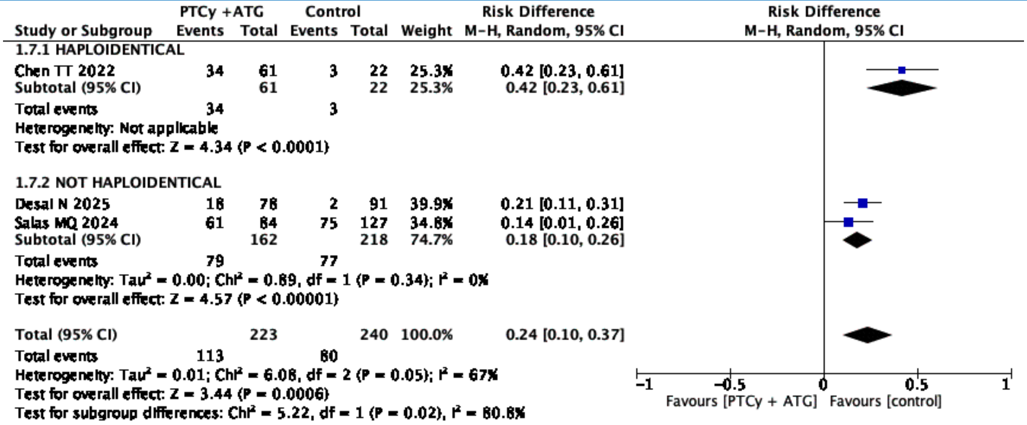

Figure 78: Analysis of EBVR: comparing PTCy plus ATG vs. other regimens
